# *In-silico* discovery of type-2 diabetes-causing host key genes that are associated with the complexity of monkeypox and repurposing common drugs

**DOI:** 10.1093/bib/bbaf215

**Published:** 2025-05-15

**Authors:** Alvira Ajadee, Sabkat Mahmud, Md Ahad Ali, Md Manir Hossain Mollah, Reaz Ahmmed, Md Nurul Haque Mollah

**Affiliations:** Bioinformatics Lab (Dry), Department of Statistics, University of Rajshahi, Rajshahi 6205, Bangladesh; Bioinformatics Lab (Dry), Department of Statistics, University of Rajshahi, Rajshahi 6205, Bangladesh; Bioinformatics Lab (Dry), Department of Statistics, University of Rajshahi, Rajshahi 6205, Bangladesh; Department of Chemistry, University of Rajshahi, Rajshahi 6205, Bangladesh; Department of Physical Sciences, Independent University Bangladesh, Bashundhara Residential Area, Dhaka 1245, Bangladesh; Bioinformatics Lab (Dry), Department of Statistics, University of Rajshahi, Rajshahi 6205, Bangladesh; Department of Biochemistry and Molecular Biology, University of Rajshahi, Rajshahi 6205, Bangladesh; Bioinformatics Lab (Dry), Department of Statistics, University of Rajshahi, Rajshahi 6205, Bangladesh

**Keywords:** type-2 diabetes, monkeypox, transcriptomics profiles and common host key genes, common drugs and toxicity, statistics and bioinformatics analysis

## Abstract

Monkeypox (Mpox) is a major global human health threat after COVID-19. Its treatment becomes complicated with type-2 diabetes (T2D). It may happen due to the influence of both disease-causing common host key genes (cHKGs). Therefore, it is necessary to explore both disease-causing cHKGs to reveal their shared pathogenetic mechanisms and candidate drugs as their common treatments without adverse side effect. This study aimed to address these issues. At first, 3 transcriptomics datasets for each of Mpox and 6 T2D datasets were analyzed and found 52 common host differentially expressed genes (cHDEGs) that can separate both T2D and Mpox patients from the control samples. Then top-ranked six cHDEGs (*HSP90AA1*, *B2M*, *IGF1R*, *ALD1HA1*, *ASS1*, and *HADHA*) were detected as the T2D-causing cHKGs that are associated with the complexity of Mpox through the protein–protein interaction network analysis. Then common pathogenetic processes between T2D and Mpox were disclosed by cHKG-set enrichment analysis with biological processes, molecular functions, cellular components and Kyoto Encyclopedia of Genes and Genomes pathways, and regulatory network analysis with transcription factors and microRNAs. Finally, cHKG-guided top-ranked three drug molecules (tecovirimat, vindoline, and brincidofovir) were recommended as the repurposable common therapeutic agents for both Mpox and T2D by molecular docking. The absorption, distribution, metabolism, excretion, and toxicity and drug-likeness analysis of these drug molecules indicated their good pharmacokinetics properties. The 100-ns molecular dynamics simulation results (root mean square deviation, root mean square fluctuation, and molecular mechanics generalized born surface area) with the top-ranked three complexes ASS1-tecovirimat, ALDH1A1-vindoline, and B2M-brincidofovir exhibited good pharmacodynamics properties. Therefore, the results provided in this article might be important resources for diagnosis and therapies of Mpox patients who are also suffering from T2D.

## Introduction

**Figure 1:**
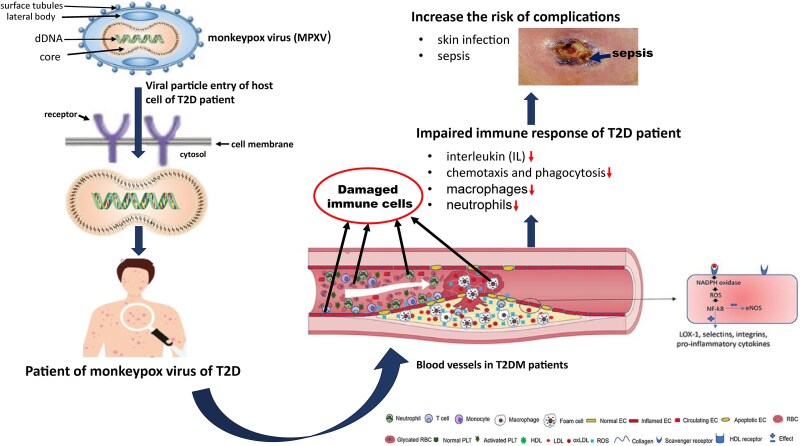
A schematic diagram about the link between T2D and Mpox infection.

Monkeypox (Mpox), a zoonotic viral disease caused by the *Orthopoxvirus* genus, was declared a public health emergency of international concern by WHO in 2022, making it a major global threat after COVID-19. It is also known as Mpox virus infection. It was first reported in 1970 that has spread worldwide, with over 99 518 cases and 207 deaths as of August 2024 [1–5]. It spreads through contact with infected animals, respiratory droplets, bodily fluids, or contaminated objects [3]. Although vaccines like JYNNEOS and ACAM2000 offer some protection, their limited availability and unawareness about Mpox underscores the urgent need for therapeutic drugs to manage it effectively [6, 7]. So there remains a critical need for therapeutic drugs against Mpox. Moreover, this infection may become more severe and complex for type-2 diabetes (T2D) patients, since T2D stimulate various types of infectious diseases including Mpox and other skin infections [8–12], due to hyperglycemia, oxidative stress, immune impairment, and altered inflammatory responses, complicating infection management [13–15]. The link between Mpox and T2D is of significant concern due to the compounded effects of immune system dysfunction and metabolic disturbances that are the characteristics of T2D patients. Mpox infection progresses in four stages—entry, fusion, replication, and release—where the virus enters host cells, releases its genetic material, replicates, and then spreads to new cells [16]. However, individuals with T2D have an inherently weakened immune system, making them more susceptible to infections like Mpox. Chronic hyperglycemia in T2D impairs the function of key immune cells, such as neutrophils and macrophages, which are responsible for initiating immune responses and clearing infections. This impairment reduces the body’s ability to mount an effective defense against pathogens, including Mpox [17, 18]. Furthermore, high blood sugar levels lead to the overproduction of reactive oxygen species, causing oxidative stress that damages endothelial cells, promotes atherosclerosis, and compromises blood flow [19, 20]. As a result, T2D patients are at an increased risk of developing severe complications, such as sepsis, where the immune system’s overreaction to infection causes widespread inflammation and tissue damage. Sepsis in T2D patients can hinder wound healing, leading to the formation of abscesses that are difficult to treat [21]. The immune dysfunction and metabolic disturbances in T2D individuals therefore result in more severe, prolonged Mpox infections, complicating treatment and management [22, 23]. Therefore, investigation of genetic link between Mpox and T2D is crucial to understand how these diseases exacerbate each other. It might be allowed for exploring common drugs for better treatment against both diseases during their co-occurrence. A schematic diagram about the link between T2D and Mpox infection is displayed in Fig. 1. The necessary data for Fig. 1 were collected from some previous studies [16–21, 24].

While numerous studies have examined the association between T2D and various infectious diseases—including COVID-19 [25, 26], influenza [27, 28], and skin infections [25, 26]—there is a notable scarcity of research investigating the mechanisms linking T2D with Mpox and exploring common drugs for the treatment of both diseases. There are several individual studies on T2D [29, 30] and Mpox [31, 32] that explored individual disease-causing host key genes (HKGs) to disclose molecular mechanisms of their development and progression individually. Although T2D exacerbates the severity of Mpox, so far, no study has explored T2D-causing common HKGs (cHKGs) that also may be associated with the complexity of Mpox. Moreover, no study has disclosed their common pathogenetic mechanisms and candidate drugs as their common treatment. On the other hand, FDA-approved drugs for T2D, such as metformin [33] and pioglitazone [34], and for Mpox, such as tecovirimat [35] and brincidofovir [36] (approved for smallpox treatment but currently available for emergency use in Mpox treatment) are already in use, but no drugs have yet been identified for the common treatment of both diseases. Therefore, doctors may need to prescribe T2D and Mpox specific multiple drugs to the patients during their co-existence. Diseases specific multiple drugs may create toxicity or adverse side effect to the patients due to drug–drug interaction [37–41]. Therefore, exploring common drugs for T2D and Mpox is essential to reduce drug burden to the patients. To explore common drugs for both T2D and Mpox, it is essential to identify both diseases causing cHKGs [42–44]. However, identifying top-ranked cHKGs and potential therapeutic agents through wet-lab experiments alone is challenging due to time-consuming, labor-intensive, and costly nature. Bioinformatics approach is promising to reduce the experimental load in wet-lab. Therefore, this study aims to explore both T2D- and Mpox-causing cHKGs to reveal shared pathogenetic mechanisms and repurposable common drugs for their treatment.

## Methodology

In order to explore repurposable candidate drugs as the common treatment for both Mpox and T2D, it is required to identify Mpox- and T2D-causing cHKGs as the targets of candidate drugs [45–48]. Transcriptomics profile analysis through bioinformatics tools is a popular approach to detect disease-causing cHKGs [49–53]. Therefore, in this study, we also considered transcriptomics profile analysis through bioinformatics tools to disclose cHKGs associated with both T2D and Mpox as introduced below.

### Data sources and descriptions

#### Transcriptomics profiles collection (case/control)

To explore T2D- and Mpox-causing cHKGs, we downloaded three host transcriptomic datasets (case/control) with accession IDs GSE36854 [54], GSE24125 [55], and GSE11234 [56] for Mpox; three datasets with accession IDs GSE19420 [57], GSE25724 [58], and GSE29226 [59]; and GSE249102 [60], GSE76895 [61], and GSE78721 [62] for T2D, from the NCBI Gene Expression Omnibus database that is publicly available. In the case of T2D, we considered three datasets from three different countries to explore more stable cHKGs for different environment, while for Mpox, we considered three datasets from two countries, since there were no other publicly available transcriptomics datasets for Mpox during our analysis. The specifics of these datasets are detailed in Table 1.

**Table 1:** Detail information of transcriptomics datasets that were analyzed in this study.

Accession IDs for datasets	Disease name	Country	Platform	Case	Control
GSE24125	Mpox	USA	GPL10912 SMD Print_1046 LC-48 *Homo sapiens*	24	28
GSE36854	Mpox	Germany	GPL4133 Agilent-014850 Whole Human Genome Microarray 4x44K G4112F	3	3
GSE11234	Mpox	USA	GPL6762 Print_1046	48	28
GSE19420	T2D	The Netherlands	GPL570 [HG-U133_Plus_2] Affymetrix Human Genome U133 Plus 2.0 Array	18	24
GSE25724	T2D	Italy	GPL96 [HG-U133A] Affymetrix Human Genome U133A Array	6	7
GSE29226	T2D	India	GPL6947 Illumina HumanHT-12 V3.0 expression BeadChip	12	12
GSE249102	T2D	Mexico	GPL570 [HG-U133_Plus_2] Affymetrix Human Genome U133 Plus 2.0 Array	8	14
GSE76895	T2D	Switzerland	GPL570 [HG-U133_Plus_2] Affymetrix Human Genoe U133 Plus 2.0 Array	36	32
GSE78721	T2D	India	GPL15207 [PrimeView] Affymetrix Human Gene Expression Array	69	61

#### Collection of candidate drug molecules

To explore cHKGs-guided top-ranked repurposable common treatment for both Mpox and T2D, we considered 215 anti-pox drugs from the DrugBank database [63], 128 cHKG-related drugs from DGIdb database [64] (Table S1), and 240 T2D-related drugs from published sources (Table S2), as the primary candidates of common drugs.

### Identification of common host differentially expressed genes

The linear models for microarray data (LIMMA) approach is a commonly used method for identification of differentially expressed genes (DEGs) between case and control groups [65]. DEGs are able to separate case group from the control group based on their expression patterns. To identify DEGs from each of T2D and Mpox host transcriptomics datasets, at first, the datasets were normalized using the robust multi-array average R-package [66] to ensure consistency across the different platforms and eliminate discrepancies. To address potential batch effects and technical variations, we applied the “Combat” function from the SVA package [67], which corrects for systematic differences between datasets. Then, we used LIMMA R-package to identify host DEGs (HDEGs) between T2D and control groups, as well as between Mpox and control groups, separately. By calculating adjusted *P*-values and log_2_fold-change (log_2_FC) values, significant HDEGs were selected for each dataset. The threshold values at adjusted *P* < .05 and log_2_FC > 1 were used to select the upregulated HDEGs and, adjusted *P* < .05 and log_2_FC < −1 to select the downregulated HDEGs. Several transcriptomics studies also used this threshold value to ensure statistical and biological significance of DEGs [31, 68–70]. Then, we calculated common upregulated HDEGs, as well as common downregulated HDEGs between Mpox and T2D. Finally, common upregulated and downregulated HDEGs were combined to construct a common HDEGs (cHDEGs) set that are able to separate both Mpox and T2D patients from the control samples. A more detailed description of the LIMMA methodology is available in Method S1.

### Common host key gene identification

The protein–protein interaction (PPI) networks of cHDEGs were constructed with two databases STRING v11.5 [71] and IMEx [72], separately to identify cHKGs. The PPI networks were visualized using Cytoscape software [73]. To prioritize cHKGs within the networks, the CytoHubba [73] plugin in Cytoscape was employed. Six topological measures—closeness, degree, maximum neighborhood component (MNC), edge percolated component (EPC), maximal clique centrality (MCC), and betweenness—were used to identify cHKGs from both databases.

### Disclosing common pathogenetic mechanisms

In order to disclose common pathogenetic mechanisms between T2D and Mpox, regulatory factors, biological processes (BPs), molecular functions (MFs), cellular components (CCs), and pathways were investigated through cHKGs as discussed in the following two subsections.

#### Identification of key regulators for common host key genes

We conducted regulatory network analysis with transcription factors (TFs) and microRNAs (miRNA) to investigate the regulators of cHKGs. In order to determine the primary TFs connected with cHKGs, we analyzed the TF–cHKG connection network with JASPAR database [74]. By examining the links between miRNA and cHKGs using the TarBase [75] databases, it was possible to identify the significant miRNAs that have an impact on cHKGs at the post-transcriptional stage. NetworkAnalyst [76] was used to replicate these interactions. The post-transcriptional regulators of cHKGs were selected from top-ranked miRNAs. We used Cytoscape [77] to visualize the networks of their interactions.

#### The common host key gene-set enrichment analysis with Gene Ontology terms and Kyoto Encyclopedia of Genes and Genomes pathways

To investigate the functional roles and pathways associated with cHKGs, we performed cHKGs-set enrichment analysis with Gene Ontology (GO) terms and Kyoto Encyclopedia of Genes and Genomes (KEGG) pathways. These analyses were conducted using three web-tools Enrichr [78], DAVID [79], and GeneCodis-4 [80]. A Fisher’s exact test was applied in each tool with a cutoff of *P*-value at .05 for statistical significance.

### The common host key gene-guided drug repurposing

To identify repurposable common drug molecules for Mpox and T2D, we conducted molecular docking to evaluate binding affinities with cHKGs. We assessed drug-likeness properties, pharmacokinetics (PK), toxicity, and performed dynamic simulations to ensure efficacy and safety as discussed in the following four subsections.

#### Molecular docking

We investigated potential drugs for both Mpox and T2D using *in silico* methods, specifically molecular docking. Molecular docking assesses drug candidates by calculating their binding affinities with target proteins, including receptors and TFs associated with cHKGs. Receptor structures were sourced from the Protein Data Bank (PDB) [81], AlphaFold [82], and SWISS-MODEL [83], while potential drug structures were obtained from PubChem [84]. The study involved preprocessing receptors using AutoDock tools [85], optimizing drug molecules with Avogadro [86], and then performing docking with AutoDock Vina [85] to determine binding affinities. Receptors and ligands were ranked based on their average binding affinity scores (BASs) to identify the top potential drug candidates for both Mpox and T2D treatment. For a detailed description of the molecular docking procedures, please see Method S2.

#### Pharmacokinetics and toxicity analysis

In drug repurposing, strong docking results do not guarantee success if a compound fails absorption, distribution, metabolism, excretion, and toxicity (ADME/T) criteria like poor absorption, rapid metabolism, or high toxicity. ADME/T analysis is crucial for selecting drug candidates with favorable PK and safety profiles, ensuring only viable options advance, especially when evaluating drug molecules for repurposing potential. ADME profiles are critical for these assessments and can often replace traditional *in vivo* and *in vitro* studies [87–90]. The ADMETlab2.0 [91] web server was used to predict the ADME features of the tested drug molecules. Additionally, toxicity properties were investigated using ProTox [92] and pkCSM [93] web tools, which assess PK and potential toxicity in the human body.

#### Drug-likeness properties

The drug-likeness profile of the collected drug molecular structure was evaluated using Lipinski’s Rule of Five (Ro5) [94], a standard method for assessing oral absorption potential and similarity to lead compounds. To determine the drug-likeness features of specific ligands, we utilized online tools such as ADMETlab 2.0 [91] and pkCSM [93]. These servers predicted various physicochemical properties (e.g. molecular weight, hydrogen bond interactions, LogP) and assessed drug-likeness and medicinal chemistry aspects, including PAINS, Brenk, and synthetic accessibility. The drug agents were provided in SMILES format, which were sourced from the PubChem database [95].

#### Molecular dynamics simulation

Molecular dynamics (MD) were employed to validate the results of molecular docking studies by assessing the stability of the top three drug-receptor complexes over a 100-ns timeframe. These simulations, conducted using the CHARMM36 force field and Gromacs 2020 software [96, 97], involved placing the complexes in a water box with TIP3P water molecules and neutralizing them with ions. Energy minimization, followed by equilibration at 310 K, was performed before trajectory analysis using metrics such as root mean square deviation (RMSD), root mean square fluctuation (RMSF), and molecular mechanics generalized born surface area (MM-GBSA) binding free energy [98]. The binding free energy (ΔGbind) was calculated using the MM-GBSA method to evaluate interaction stability. Trajectory files were analyzed with Gromacs and VMD, and data were converted to GROMACS-compatible formats with InterMol software. For a detailed description of the Molecular dynamic’s simulation procedures, please see Method S2.

**Figure 2:**
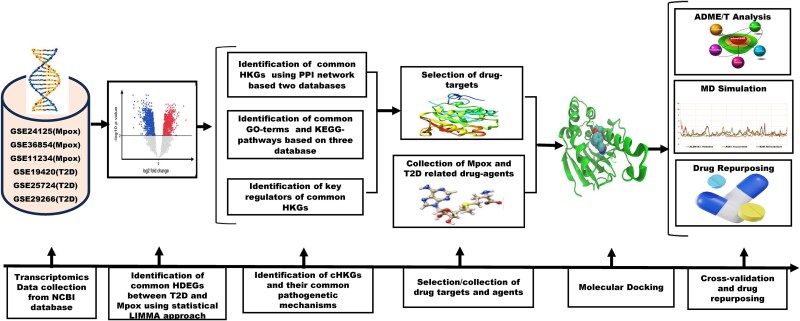
A graphical representation of the entire workflow.

A comprehensive graphical representation of the entire workflow (data collection, DEG identification, PPI network construction, and drug repurposing) is provided in Fig. 2.

## Results

### Identification of common host differentially expressed genes

The genes with differential expression between Mpox/T2D and control samples were obtained from each of the six datasets through statistical LIMMA method. We analyzed gene expression data from three datasets each of Mpox and T2D. For Mpox, 518 common HDEGs (86 upregulated, 432 downregulated) were identified from 1617, 1255, and 1958 upregulated and 7097, 3345, and 6432 downregulated HDEGs across datasets GSE11234, GSE36854, and GSE24125, respectively (Table S3). In T2D, 159 common HDEGs (73 upregulated, 86 downregulated) were identified from 2003, 2242, 1612, 2256, 1536 and 2359 upregulated and 1097, 2756, 5412 1322, 912, and 1652 downregulated DEGs across datasets GSE19420, GSE25724, GSE29226 GSE249102, GSE76895, and GSE78721, respectively (Table S3). Comparing both conditions, 32 upregulated and 20 downregulated common HDEGs (cHDEGs) were identified (Table S4).

### Common host key gene identification

**Figure 3:**
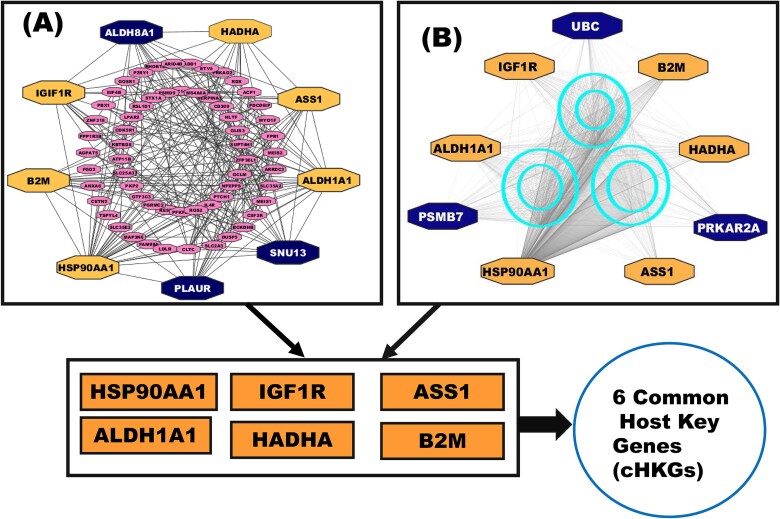
PPI networks based on (A) STRING and (B) IMEx databases. In both A and B, larger nodes indicate the top-ranked cHDEGs, where brown nodes in both A and B represent the T2D- and Mpox-causing cHKGs in both databases.

To identify cHKGs, we constructed two separate PPI networks for the cHDEGs: one based on the STRING database and the other based on the IMEx database. To determine genes with high connectivity, which are crucial for network stability and biological function, we employed six topological analysis methods: bottleneck, closeness, degree, EPC, MCC, and MNC. In the STRING database-based PPI network, we identified nine top-ranked cHDEGs with the highest connectivity (HSP90AA1, ALDH1A1, SNU13, IGF1R, B2M, ALDH8A1, HADHA, ASS1, PLAUR). Similarly, in the IMEx-based PPI network, nine top-ranked cHDEGs were identified (HSP90AA1, PSMB7, B2M, ASS1, IGF1R, HADHA, UBC, ALDH1A1, and PRKAR2A). Finally, six cHDEGs (HSP90AA1, B2M, IGF1R, ALD1HA1, ASS1, and HADHA) were considered as cHKGs that are common between these two database-based top-ranked cHDEG sets. These cHKGs are highlighted in Fig. 3 and detailed in Tables S5–S7.

### Disclosing common pathogenetic mechanisms

In order to disclose common pathogenetic mechanisms between T2D and Mpox, regulatory factors, BPs, MFs, CCs, and pathways were investigated through cHKGs as discussed in the following two subsections.

#### Identification of key regulators for cHKGs

**Figure 4:**
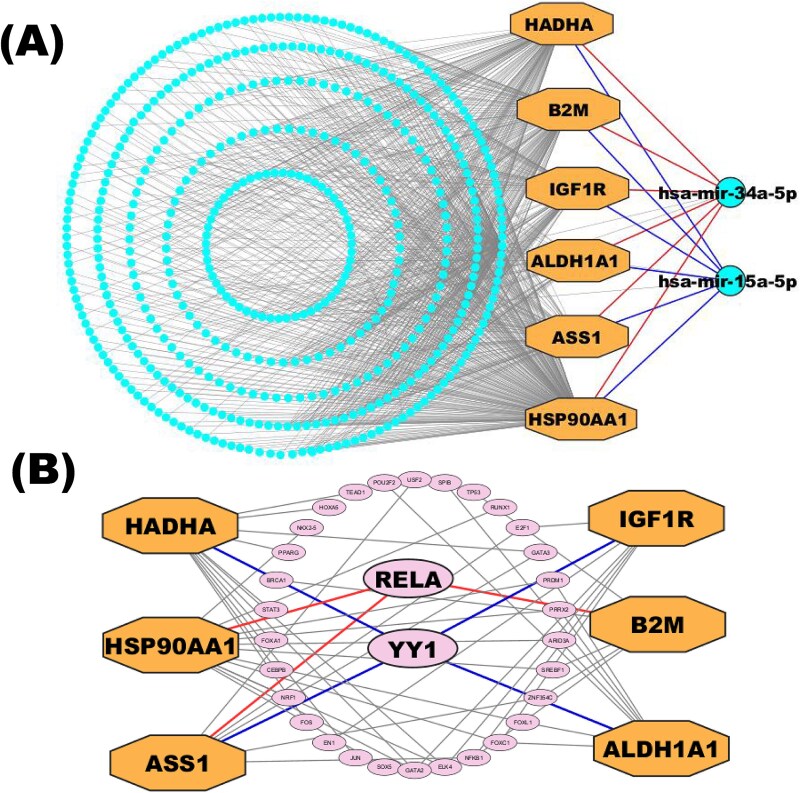
The interaction network visualized the relationships between (A) miRNAs and cHKGs and (B) TFs and cHKGs, where larger octagonal nodes indicate cHKGs in both A and B. Two larger circular nodes in A and two larger oval nodes in B indicated by the top-ranked miRNAs and TFs, respectively, represent the key regulators of cHKGs.

To find the cHKGs transcriptional and post-transcriptional regulators, we looked into the networks of TFs and miRNAs with cHKGs. In the beginning, we chose the top two transcriptional regulators of cHKGs (RELA and YY1) based on two topological measures with cutoff degrees of 4 and betweenness of 275.42. Then, using the same topological measures with cutoff degrees of 6 and betweenness of 755.75, We detected top ranked two miRNAs (hsa-mir-34a-5p and hsa-mir-15a-5p) to serve as post-transcriptional regulators of cHKGs (Fig. 4).

#### The common host key gene-set enrichment analysis with Gene Ontology terms and Kyoto Encyclopedia of Genes and Genomes pathways

The GO and KEGG pathway analysis of the six cHKGs revealed overlapping biological mechanisms between Mpox and T2D. We first performed cHKGs-set enrichment analysis using the Enrichr [78] and validated the results by identifying GO terms and KEGG pathways that were also significantly enriched in DAVID [79] and GeneCodis4 [80] web tools. This process identified four significant BP terms, four CC terms, three MF terms, and two KEGG pathways. These results, detailed in Table 2 and Tables S8–S10, highlight critical biological activities and pathways shared by the two databases, providing insights into potential therapeutic targets.

**Table 2:** The top significantly (*P* < .05) enriched GO terms and KEGG pathways with cHKGs in at least two databases out of three (Enrichr, DAVID, and Genecodies).

Annotation ID	BP	Agreed databases	Associated cHKGs
GO:0042026	Protein refolding	Enrichr, DAVID	HSP90AA1, B2M
GO:0045429	Positive regulation of nitric oxide biosynthetic process	DAVID, Genecodies	HSP90AA1, ASS1
GO:0001934	Positive regulation of protein phosphorylation	Enrichr, DAVID	HSP90AA1, ALDH1A1
GO:0001889	Liver development	Enrichr, DAVID	ASS1
**Annotation ID**	**MF**	**Agreed databases**	**Associated cHKGs**
GO:0004029	Disordered domain specific binding	Enrichr, DAVID, Genecodies	HSP90AA1
GO:0023026	MHC class II protein complex binding	Enrichr, DAVID, Genecodies	HSP90AA1, B2M
GO:0005009	Insulin receptor activity	Enrichr, DAVID	IGFR1
GO:0042803	Protein homodimerization activity	DAVID, Genecodies	HSP90AA1, B2M
**Annotation ID**	**CC**	**Agreed databases**	**Associated cHKGs**
GO:0042470	Melanosome	Enrichr, DAVID	HSP90AA1
GO:0070062	Extracellular exosome	Enrichr, DAVID	HSP90AA1, B2M, ASS1, HADHA
GO:0005925	Focal adhesion	Enrichr, DAVID, Genecodies	ALDH1A1, B2M, HADHA
GO:0005829	Cytosol	Enrichr, DAVID	HSP90AA1, B2M, ASS1, HADHA
**Annotation ID**	**KEGG pathway**	**Agreed databases**	**Associated cHKGs**
hsa01230	Biosynthesis of amino acids	Enrichr, DAVID	ASS1
hsa04612	Antigen processing and presentation	Enrichr, DAVID, Genecodies	HSP90AA1, B2M
hsa04914	Progesterone-mediated oocyte maturation	Enrichr, DAVID, Genecodies	HSP90AA1; IGF1R

### The common host key gene-guided drug repurposing

To identify repurposable common drug molecules for Mpox and T2D, we conducted molecular docking to evaluate binding affinities with cHKGs. We assessed drug-likeness properties, PK, and toxicity and performed dynamic simulations to ensure efficacy and safety as discussed in the following four subsections.

#### Molecular docking

**Figure 5:**
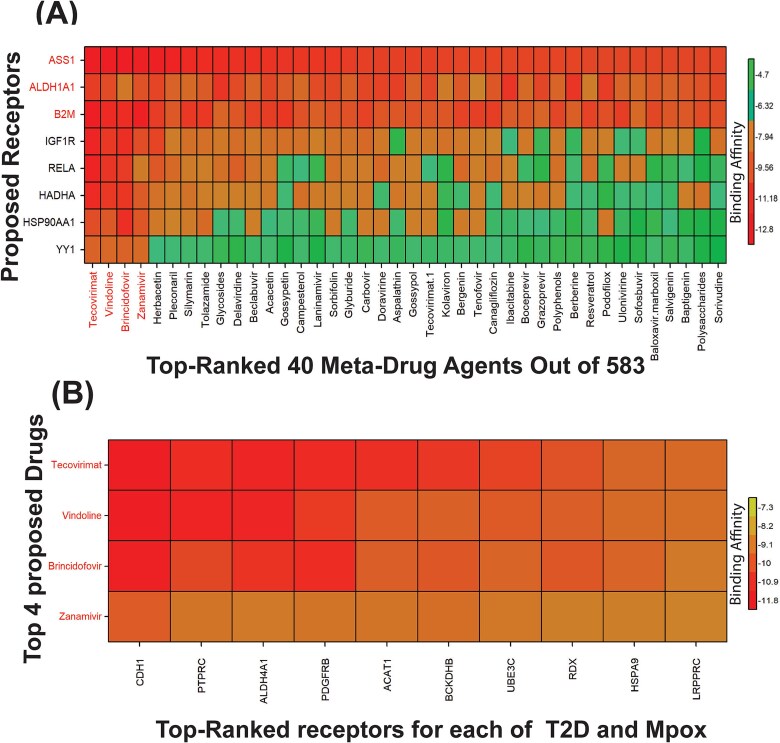
Image of drug-target binding affinity matrices. (A) X-axis indicates top-ordered 40 drug agents (out of 583) and Y-axis indicates ordered proposed receptor proteins. (B) X-axis shows top-ranked five Mpox-causing proteins and top-ranked five T2D-causing proteins, and Y-axis indicates top 4 proposed drug agents as the common treatment for both T2D and Mpox.

To explore candidate common drug molecules for both T2D and Mpox, we have considered cHKGs-mediated six common host key proteins and their two TF proteins as receptor proteins. Among these, the PDB provided the 3D structures of eight proteins (HSP90AA1, B2M, IGF1R, ALD1HA1, ASS1, HADHA, RELA, and YY1) with 2bug, 4wb9, 1JQH, 1ywh, 6DV2, 2lsp, and 1znm PBD codes, respectively. The 3D structure of ACO2 was obtained using the “AlphaFold Protein Structure Database” using UniProt with ID Q99798. BASs were determined through molecular docking analysis between the proposed receptors and meta-drug compounds. These scores measure the strength of interaction between two molecules, such as a drug and its target protein, with lower scores (typically in kcal/mol) indicating stronger and more stable binding interactions. These scores are crucial in drug discovery, as they help predict a compound’s potential effectiveness in modulating a target’s activity. Then, we selected a small number of drug agents as the candidate drugs by sorting the target receptors in descending order of the row sums of the binding affinity matrix B = (S*_ij_*) and drug agents based on the column sums (Fig. 5; Table S11). The study identified significant binding affinities (BAS ≤ 7.0 kcal/mol) between all 10 receptor proteins and the top four lead drugs (tecovirimat, vindoline, brincidofovir, and zanamivir). Then we investigated their binding performance against the top-ranked Mpox-causing eight HKPs (B2M, CDH1, HSP90AA1, PTPRC, IGF1, PLAUR, PDGFRB, and ASS1), as well as T2D-causing nine HKPs (HSP90AA1, ASS1, HADHA, UBE3C, ACAT1, HSPA9, ALDH4A1, LRPPRC, and RDX) that were identified through PPI network analysis of their individual DEGs-sets (Fig. S1; Table S3). We observed that T2D-causing top-ranked six HKPs (UBE3C, ACAT1, HSPA9, ALDH4A1, LRPPRC, and RDX) and Mpox-causing top-ranked four HKPs (CDH1, PTPRC, IGF1, and PDGFRB) does not belong to cHKG set (Fig. S1). By performing molecular docking analysis between these uncommon proteins with the selected drug compound, we found that their BAS ≤ 7.0 kcal/mol, which indicates their significant binding performance (Table S12). Additionally, we examined the drug-target binding positions using AutoDock Vina, with 2D schematic diagrams of receptor–ligand interactions and 3D views of the complexes provided in Table 3, highlighting interacting residues.

**Table 3:** Top-ranked three 3 drug-target complexes highlighting their 3-dimension (3D) view and interacting residues.

Protein and ligand complex	Binding affinity (kcal/ mol)	The 3D view of complexes	Target–ligand interaction highlighting targeted residues
ASS1 and tecovirimat	−12.2	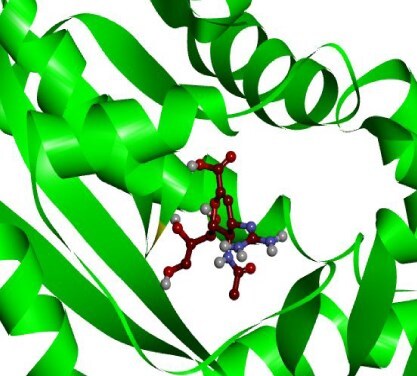	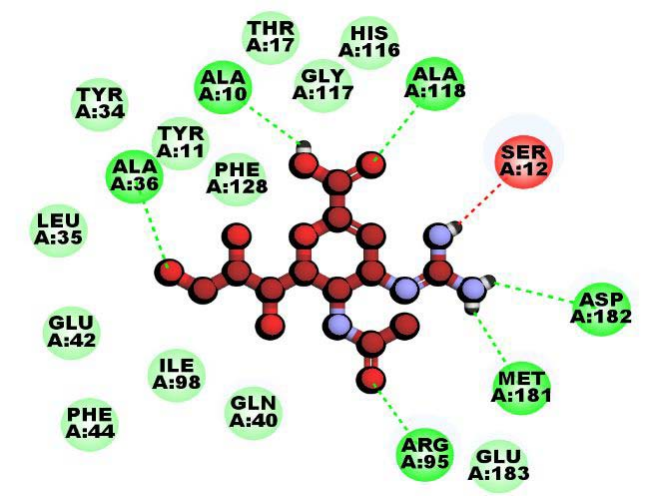
ALDH1A1 and vindoline	−11.5	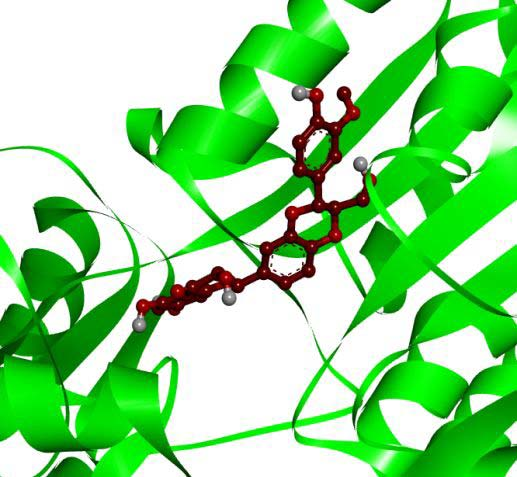	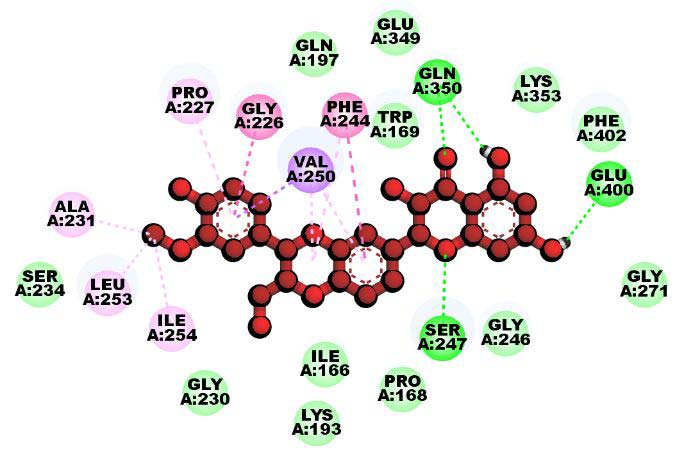
B2M and brincidofovir	−11	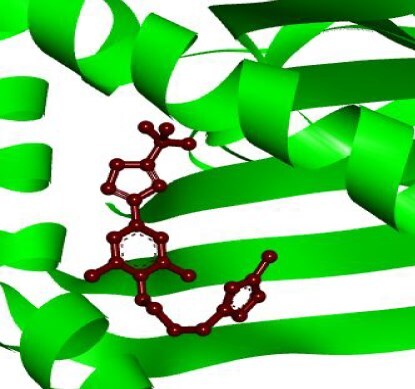	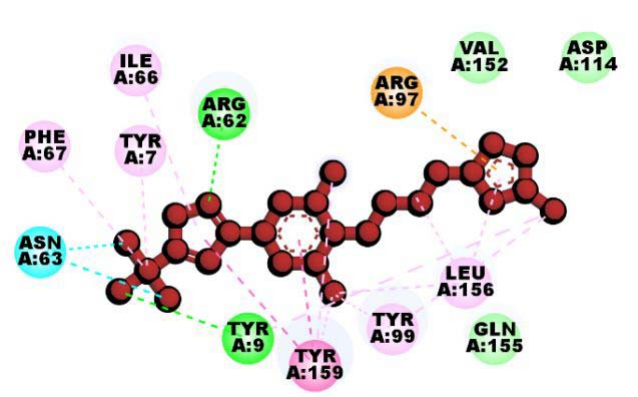

#### Pharmacokinetics and toxicity analysis

The ADME/T properties of a drug molecule help determine its suitability for use. Drug-likeness evaluates its physicochemical characteristics (Table 4). For oral medications, effective absorption in the gastrointestinal tract is essential. A compound is considered well-absorbed if its human intestinal absorption (HIA) score is >30% [93, 99]. In our study, all drug molecules except zanamivir had high HIA scores (≥68%), indicating strong absorption. Caco-2 permeability predicts gastrointestinal absorption, with values ≥5.15 log cm/s considered adequate [93, 100]. Experimental data suggest that all top-ranked compounds are well absorbed in the gut. The blood–brain barrier (BBB) permeability index assesses a drug’s ability to cross the BBB. Compounds with LogBB values ≤1 have poor BBB penetration, while those ≥0.3 can cross it effectively [101]. None of the suggested drugs crossed the BBB effectively, as all had LogBB values below 0.3 (Table 4). Volume of distribution (VDss) indicates how well a drug spreads in the plasma. Values above 2.81 l/kg are considered high [99], and our compounds showed excellent VDss, meaning good plasma distribution. All compounds are also evaluated for central nervous system (CNS) penetration based on LogPS (CNS) values. Our compounds were predicted to partially reach the CNS. Cytochrome P450 (CYP) enzymes, crucial for drug metabolism, interact with many drugs [102]. The compounds showed selective CYP interactions, suggesting potential for drug repurposing. The excretion profile of these compounds indicates they are effectively removed from the bloodstream, with total clearance (TC) values between 1 and 5 l/h, suggesting minimal risk of accumulation. Toxicity analysis, including AMES testing, shows no mutagenicity for the selected drug agents, and they also do not cause skin sensitization, addressing key safety concerns. These findings confirm that the drug agents possess favorable pharmacokinetic properties and are unlikely to cause harm or adverse effects. Consequently, their therapeutic potential against both Mpox and T2D is supported by their favorable *in silico* profiles.

**Table 4:** ADME/T analysis of selected top-ranked four candidate drug molecules using different web tools.

Compounds	Absorption			Distribution		Metabolism (CYP450)				Toxicity				
	Caco2	LogS	HIA (%)	BBB	VDss	CYP1A2 Inhibitor	CYP2C19 inhibitor	CYP2C9 Substrate	CYP2D6 inhibitor	TC	AMES Toxicity	Rat Oral Acute Toxicity	Skin Sensitization	Carcinogenicity
Tecovirimat	0.87	−4.65	92.812	0.116	0.884	No	No	No	No	1.83	No	0.306	0.079	No
Brincidofovir	−0.05	−2.94	70.031	0.039	0.746	No	No	No	No	2.44	No	0.004	0.916	No
Vindoline	0.22	−4.43	96.576	0.261	1.846	No	No	No	Yes	2.07	No	0.027	0.051	No
Zanamivir	5.11	−0.563	21.234	0.039	0.801	Yes	No	No	Yes	7.11	No	0.025	0.024	No

#### Drug-likeness properties

After molecular docking, the top-ranked drug molecules (tecovirimat, vindoline, brincidofovir, and zanamivir) were assessed for drug-likeness. They showed strong pharmacokinetic properties with minimal violations of Lipinski’s Ro5. All compounds met key criteria and also followed Veber’s and Egan’s rules, except zanamivir (Table S13). All drugs support their potential for drug repurposing. For an in-depth explanation, please see Result S1.

#### Molecular dynamics analysis

**Figure 6:**
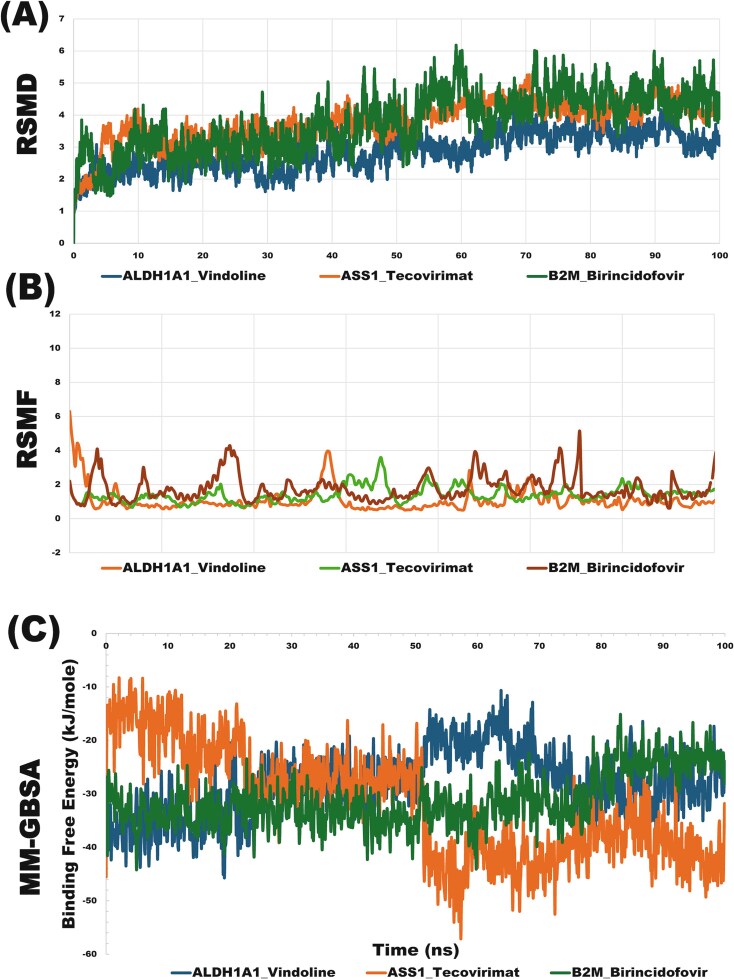
(A) The RMSD analysis results for a duration of 100-ns simulation with each of the top-ranked three drug-target complexes, (B) the RMSF analysis results for a duration of 100-ns simulation with each of the top-ranked three drug-target complexes, and (C) binding free energy (MM-GBSA) calculations of the top-ranked three drug-target complexes.

Following the molecular docking study, the top three proteins–ligands complexes were subjected to 100-ns MD simulations to assess their stability and behavior. The analysis involved monitoring key parameters such as RMSD, RMSF, and MM-GBSA binding free energy. RMSD measures the average displacement of atoms over time compared to a reference structure, reflecting the overall stability of the protein–ligand complex. The average RMSD values for the ALDH1A1-vindoline, ASS1-tecovirimat, and B2M-brincidofovir complexes stabilized at 2.84, 3.77, and 3.78 Å, respectively, indicating moderate structural deviations and relatively stable configurations. ALDH1A1-vindoline had the lowest RMSD, suggesting stronger binding and enhanced stability compared to the other complexes (Fig. 6A). RMSF quantifies the flexibility of individual amino acid residues in the protein, highlighting regions with significant fluctuations. The RMSF analysis indicates that the ALDH1A1-vindoline complex has the highest rigidity and stability, with an average value of 1.09 Å, compared to 1.41 Å for ASS1-tecovirimat and 1.75 Å for B2M-brincidofovir (Fig. 6B). This suggests that ALDH1A1-vindoline forms the most consistent interaction with its target. MM-GBSA binding free energy calculations estimate the binding energy of the protein–ligand complex, with more negative ΔG values indicating stronger binding. Using the Desmond module of Schrödinger, the average ΔG values were −28.06 kJ/mol for ALDH1A1-vindoline, −31.93 kJ/mol for ASS1-tecovirimat, and −31.21 kJ/mol for B2M-brincidofovir, indicating significant stability. ASS1-tecovirimat showed the strongest interaction, followed by B2M-brincidofovir and ALDH1A1-vindoline (Fig. 6C). These results suggest that tecovirimat, brincidofovir, and vindoline exhibit stable and effective binding to their target proteins, highlighting their potential as therapeutic agents for treating both Mpox and T2D.

## Discussion

Mpox is a viral disease that has recently emerged as a significant health concern, especially in individuals with pre-existing comorbidities [103]. Among these comorbidities, T2D has emerged as a potential risk factor for more severe outcomes in viral infections like Mpox [12]. Transcriptomic analyses have proven to be a powerful tool in the study of various diseases, including autoimmune disorders, viral infections, cancer, and neurodegenerative conditions [104–108]. Using transcriptomic analyses, we identified six shared key host genes (*HSP90AA1*, *B2M*, *IGF1R*, *ALD1HA1*, *ASS1*, and *HADHA*) significantly enriched in important BPs (GO terms) and pathways (KEGG), shedding light on their potential roles in the comorbidity between these two diseases. Among our identified cHKGs, *HSP90AA1* is critical for *protein refolding* and *MHC class II binding*, essential for cellular homeostasis and immune regulation [109]. Dysregulation of *HSP90AA1* in T2D has been linked to impaired insulin signaling, contributing to metabolic dysfunction and inflammatory pathways [110]. In Mpox, it may facilitate viral replication by stabilizing viral proteins and modulating host immune responses, enhancing viral persistence. The gene’s involvement in protein homodimerization activity and RNA binding highlights its importance in maintaining protein stability and regulating gene expression, both of which are disrupted during inflammation and viral infections [111]. *HSP90AA1*’s role is critical in regulating immune responses and metabolic balance, both of which are compromised in T2D and Mpox infection. *B2M* is a critical component of the MHC class I complex, which plays a vital role in antigen presentation and immune surveillance. In the context of T2D, hyperglycemia-induced oxidative stress elevates its levels, driving chronic inflammation and metabolic dysfunction. During Mpox infection, B2M may contribute to immune evasion by disrupting antigen presentation, weakening antiviral responses [112]. This dual role highlights the importance of *B2M* as both a marker of immune dysregulation and a potential contributor to poor outcomes in Mpox patients with pre-existing T2D. *ALDH1A1* metabolizes retinaldehyde to retinoic acid, essential for glucose and lipid regulation. In T2D, reduced ALDH1A1 activity worsens glucose intolerance and insulin resistance [113, 114]. During Mpox infection, the virus-induced oxidative stress could overwhelm ALDHA1’s ability to neutralize reactive aldehydes, leading to increased cellular damage [115, 116]. HADHA is integral to mitochondrial fatty acid β-oxidation, facilitating the final steps of long-chain fatty acid metabolism. In T2D, impaired HADHA function can disrupt fatty acid metabolism, potentially contributing to insulin resistance and metabolic disturbances [117]. In Mpox, its role in energy metabolism and immune response modulation might influence disease severity [118]. ASS1 is crucial for nitric oxide biosynthesis and amino acid metabolism, supporting vascular health and metabolic balance [119]. In T2D, reduced ASS1 expression impairs nitric oxide production, causing endothelial dysfunction and complications like atherosclerosis and diabetic nephropathy [120]. Mpox disrupts *ASS1* function, hindering immune responses and tissue repair, amplifying metabolic instability and disease severity [121]. IGF1R mediates IGF-1 effects, influencing growth and metabolism. In T2D, damaging missense variants in IGF1R lead to IGF-1 resistance, characterized by shorter stature and elevated IGF-1 levels [122]. In Mpox, its role in cell survival and immune response influence disease severity [123]. Thus, the dysregulation of these cHKGs significantly contributes to disease progression, intensifying the severity of comorbid conditions. The regulatory analysis highlighted the involvement of two TFs (RELA and YY1) and two miRNAs (hsa-mir-34a-5p and hsa-mir-15a-5p) in modulating the expression of cHKGs between Mpox and T2D (Fig. 4). All cHKGs are regulated by RELA and YY1, which profoundly influences their expression across various physiological and pathological conditions. RELA, a critical component of the NF-κB complex, plays an essential role in orchestrating inflammatory responses and stress signaling pathways [124]. Concurrently, YY1, a versatile TF, modulates genes involved in stress responses, metabolic processes, and immune functions [125]. In addition, miRNAs such as hsa-mir-34a-5p and hsa-mir-15a-5p, which are involved in the regulation of gene expression at the post-transcriptional level, have been shown to play roles in both viral infections and metabolic diseases. hsa-mir-34a-5p has been linked to the regulation of inflammation, apoptosis, and oxidative stress—all of which are central to the pathogenesis of T2D and Mpox [126]. Similarly, hsa-mir-15a-5p has been implicated in regulating insulin sensitivity and inflammatory responses, further supporting its potential role in the pathophysiology of T2D and viral infections [127, 128].

In order to explore candidate drugs against Mpox, we may consider pathogenic proteins of Mpox [129–133] or infection-causing dysregulating host receptor proteins/genes [134] as the drug targets, since pathogenic proteins interact with the host proteins to develop infection [135–140]. This study considered both T2D- and infection-causing cHKGs-guided drug repurposing against both diseases. We performed molecular docking with 583 compounds and cHKGs-mediated proteins and identified four (tecovirimat, vindoline, brincidofovir, zanamivir) with strong binding affinities. Then tecovirimat, brincidofovir, and vindoline were carefully selected as promising candidates for treating Mpox in T2D patients through drug-likeness, PK, and toxicity evaluations to ensure their potential suitability for Mpox in T2D patients, zanamivir was excluded due to not satisfies all pharmacokinetic properties, affecting its safety and efficacy. Among them, tecovirimat, an FDA-approved (DB12020) antiviral for smallpox, targets the viral protein and demonstrates strong potential for repurposing against Mpox [141]. Brincidofovir, also FDA-approved (DB12151) antiviral for smallpox, is a lipid conjugate of cidofovir with broad-spectrum antiviral activity [142]. Tecovirimat and brincidofovir are FDA-approved antivirals but need further evaluation for safety and efficacy in T2D patients with Mpox. Vindoline—a compound with hypoglycemic, anti-inflammatory, and antiviral properties—may help improve β-cell dysfunction in T2D. This suggests its potential use in managing diabetes and related complications, contributing to the therapeutic effects of *Canthaxanthins roseus* [143]. Vindoline, though not traditionally used for Mpox, may potentially be explored for its anti-inflammatory properties to address complications like chronic inflammation in Mpox patients with T2D, though further investigation is needed for its direct antiviral effects [144]. The mechanisms of these drugs suggest a promising therapeutic approach for Mpox in T2D patients. However, though *in silico* methods including molecular docking, ADMET profiling, and toxicity analysis provide valuable insights on the proposed drug molecules, further experimental validations are required before going to clinical trials. To address this, drug cytotoxicity should first be assessed *in vitro* using host samples, ensuring proper medium preparation for accurate experimental conditions [145]. Following this, *in vivo* and *ex vivo* studies with animal models, such as Swiss albino mice, can evaluate the efficacy and safety of the top-ranked drugs in a biological context [146]. Additionally, gene expression analysis through quantitative PCR will help confirm the effects of the drugs on key target proteins identified in this study [147].

## Limitations of this study

This study analyzed independent datasets on T2D and Mpox to explore both diseases causing cHKGs, since we did not find any transcriptomics dataset from online databases that is generated from the patients who are suffering from both diseases during this study. In this study, cHKGs were selected from cHDEGs by PPI network analysis with different topological features. However, sometimes topological feature-based PPI network analysis may produce false positive and/or false negative cHKGs [148]. That is why drug screening by molecular docking with cHKG proteins may also detect some false positive and/or false negative drug molecules. Therefore, further experimental validation is required on the findings of this study.

## Conclusion

These study results indicated that T2D is associated with the complexity of Mpox. It identified both disease-causing top-ranked six common host key genes (cHKGs) *HSP90AA1*, *B2M*, *IGF1R*, *ALD1HA1*, *ASS1*, and *HADHA* that might be associated with the complexity of Mpox, through transcriptomics profile analysis. The cHKGs set enrichment analysis with BPs, MFs, CCs, KEGG pathways, and regulatory factors (TF proteins and miRNAs) disclosed shared pathogenetic mechanisms. Finally, cHKG-guided top-ranked three repurposable drug candidates (tecovirimat, vindoline, and brincidofovir) were recommended as the common treatment for both T2D and Mpox. These drug molecules might be useful to reduce the drug burden to the Mpox patients with T2D as comorbidity, since diseases specific multiple drugs may create toxicity or adverse side effect to the patients due to drug–drug interaction. However, further experimental validation is required in order to confirm the effectiveness of our findings.

**Key Points**

- Type-2 diabetes (T2D) is considered as the risk factor for the complexity of monkeypox (Mpox) through their common genetic factors.
- Identification of both disease-causing common host key genes (cHKGs) is essential for exploring candidate drugs as the common therapies for both diseases to reduce drug burden to the patients.
- This study identified both Mpox and T2D causing six cHKGs through transcriptomics profiles analysis using bioinformatics tools and databases.
- Shared pathogenetic mechanisms of T2D and Mpox were disclosed through cHKG-set enrichment analysis with Gene Ontology terms, Kyoto Encyclopedia of Genes and Genomes pathways, and regulatory networks.
- cHKG-guided three candidate drug molecules were suggested for taking a common treatment plan against Mpox patients who are also suffering from T2D.

## Supplementary Material

SupplimentaryFileBIB_bbaf215

## Data Availability

The datasets analyzed in this study were downloaded from NCBI database with the following links: https://www.ncbi.nlm.nih.gov/geo/query/acc.cgi?acc=GSE24125 https://www.ncbi.nlm.nih.gov/geo/query/acc.cgi?acc=GSE36854 https://www.ncbi.nlm.nih.gov/geo/query/acc.cgi?acc=GSE11234 https://www.ncbi.nlm.nih.gov/geo/query/acc.cgi?acc=GSE19420 https://www.ncbi.nlm.nih.gov/geo/query/acc.cgi?acc=GSE25724 https://www.ncbi.nlm.nih.gov/geo/query/acc.cgi?acc=GSE29226 https://www.ncbi.nlm.nih.gov/geo/query/acc.cgi?acc=GSE249102 https://www.ncbi.nlm.nih.gov/geo/query/acc.cgi?acc=GSE76895 https://www.ncbi.nlm.nih.gov/geo/query/acc.cgi?acc=GSE78721
