# Supplementary material for: In-silico discovery of type-2 diabetes-causing host key genes that are associated with the complexity of monkeypox and repurposing common drugs
Source: Brief Bioinform. 2025 May 15;26(3):bbaf215. doi: 10.1093/bib/bbaf215 (PMC12078936; doi:10.1093/bib/bbaf215)
Supplement: SupplimentaryFileBIB_bbaf215 [file supplimentaryfilebib_bbaf215.docx]

**Supplementary file**

***In-Silico* Discovery of Type-2 Diabetes-Causing Host Key-Genes that are Associated with the Complexity of Monkeypox, and Repurposing Common Drugs**

| **Supporting Items** | **Pages** |
| --- | --- |
| **Supplementary Methods** | |
| **Method S1.** LIMMA approach | 2-3 |
| **Method S1.** Molecular docking | 3-4 |
| **Method S3.** Molecular dynamics simulation | 4-5 |
| **Supplementary Results** | |
| **Results S1:** Drug-likeness properties | 6 |
| **Supplementary Tables** | |
| **Table S1**. Collection of drug agents for Monkeypox, smallpox and cHKGs guided drugs agents | 6-9 |
| **Table S2.** Collection of type-2 diabetes (T2D) related candidate drug agents from published articles and other sources. | 9-11 |
| **Table S3**. List of HDEGs for each of Mpox and T2D | 11-12 |
| **Table S4.** List of upregulated and downregulated cHDEGs between Mpox and T2D | 12 |
| **Table S5.** Different topological measure score of common host key genes (cHKGs) from PPI network based on STRING database. | 12-13 |
| **Table S6.** Different topological measure score of common host key genes (cHKGs) from PPI network based on IMEx database. | 13 |
| **Table S7.** Average rank of common 6 cHKGs between STRING and IMEx databases | 14 |
| **Table S8.** The top significantly (*p*-value<0.05) enriched GO terms and KEGG pathways with cHKGs by David web tool | 14-15 |
| **Table S9.** The top significantly ( *p*-value<0.05) enriched GO terms and KEGG pathways with cHKGs by GeneCodis web tool | 15-16 |
| **Table S10.** The top significantly ( *p*-value<0.05) enriched GO terms and KEGG pathways with cHKGs by GeneCodis web tool | 16-17 |
| **Table S11.** Docking/(binding affinity) scores (kcal/mol) between the proposed target genes/proteins (receptors) and top ordered 40 candidate drugs (out of 483). | 18 |
| **Table S12.** Docking/(binding affinity) scores (kcal/mol) between the individual disease-causing proteins (receptors) and top ordered 4 candidate drugs (Out of 483) | 19 |
| **Table S13.** Drug likeness & pharmacokinetics properties of top ranked 4 candidate drug molecules | 19 |
| **Supplementary Figures** | |
| **Figure S1:** Protein-protein interactions (PPIs) of HDEGs for each of Mpox and T2D | 20 |
| **References** | 21-27 |

**Supplementary Methods**

**Method S1. LIMMA approach**

The statistical linear models for microarray (LIMMA) data analysis is a widely used approach [1] for identifying differentially expressed genes (DEGs) between mpox and control samples, as well as between T2D and control samples. The LIMMA model is written as,

$z_{g}={X\alpha}_{g}+\epsilon_{g}$ _(1)_

where *z_g_* = (z*_g1_*, z*_g2_*, . . ., z*_gn_*) ^is^ the responses (expressions) *m*-vector of *g*th gene with *n* = *n*_1_+*n*_2_ samples (*g* = 1, 2, . . ., G), X is an *n*×2 design matrix, α_g_ = (α_g1_, α_g2_) ^/^ is a 2-vector (2<n) of effects for MPOX and control groups/ T2D and control groups of n samples and the error vector ϵ_g_~ N (0, W_g_ϭ_g_^2^). The *g*th gene is said to be equally expressed gene (EEG) if the null hypothesis (H_0_): α*_g_*_1_ =α*_g_*_2_ = > γ*_g_* = (α*_g_*_1_−α*_g_*_2_) = 0 is true. Otherwise, it is said to be differentially expressed gene (DEG). To test the significance of H_0_, the test statistic for LIMMA approach is defined as

$\tilde{t}_{g}=\frac{\hat{\gamma}_{g}+\gamma_{g}}{\tilde{S}_{g}\sqrt{\delta}_{g}}$ (2)

which follows *t*-distribution under H_0_. Adjusted *P*-values based on the moderated t-statistic and the average

of log_2_ fold-change (aLog_2_FC) values of treatment group with respect to the control group were used to select DEGs as follows,

$$\mathrm{DEG}_{g}=\left\{ \begin{matrix} \mathrm{DEG}\left( \mathrm{Up} \right), if adj.p.\mathrm{value}<0.05 and a\mathrm{Log}_{2}{FC}_{g}>+1.0 \\ \\ \mathrm{DEG}\left( \mathrm{Down} \right),if adj.p.\mathrm{value}<0.05 and a\mathrm{Log}_{2}{FC}_{g}<-1.0 \end{matrix} \right.$$

where,

$${aLog}_{2}{FC}_{g}=\left\{ \begin{matrix} \frac{1}{n_{1}}\sum_{i}^{n_{1}} {log}_{2}{(z}_{gi}^{T})-\frac{1}{n_{2}}\sum_{j}^{n_{2}} {log}_{2}{(z}_{gj}^{C} ), if n_{1}\neq n_{2} \\ \frac{1}{n}\sum_{i}^{n} {log}_{2}\left( \frac{z_{gi}^{T}}{z_{gj}^{C}} \right), if n_{1}=n_{2} =n \end{matrix} \right.$$

Here $z_{gi}^{T}$ and $z_{gj}^{C}$ are the responses/expressions for the *g*th gene with the *i*th case and *j*th control samples, respectively. We applied LIMMA R-package [2] for computing *P*-values and aLog2FC values to separate the DEGs/EEGs from each of six datasets, significantly. The common DEGs were filtered from three DEG sets to identify genes associated with mpox, and the same process was applied to another three DEG sets for T2D.

**Method S2. Molecular docking**

There are two primary in-silico methods for drug discovery: de-novo design, which is time-consuming and expensive, and drug repurposing (DR), which leverages existing, approved drugs for new diseases. Both methods utilize molecular docking to assess potential drug candidates by calculating their binding affinities and interactions with target proteins. Molecular docking is a widely used technique for screening potential drug molecules from a vast array of options to inhibit disease-causing genes [3,4]. Therefore, in this study, we also used this analysis to investigate KGs-guided potential therapeutic molecules/ligands for the treatment of ccRCC. For the purposes of docking study, we considered KGs-mediated receptor proteins and the regulatory TFs proteins that accompany them. The Protein Data Bank (PDB) [5], AlphaFold Protein Structure Database [6], and SWISS-MODEL [7] were the sources from which the three-dimensional (3D) receptor structures were obtained. The 3D structures of possible medicinal agents were obtained by searching the PubChem database [8]. The 3D architectures of protein interactions were visualized using the Discovery Studio Visualizer [9]. AutoDock tools were used to pre-process the receptor proteins, which involved removing water molecules and adding charges [10]. The Avogadro [11] was used to reduce the energy of the drugs agents, and AutoDock tools [10] were used to preprocess them. After that, AutoDock Vina was used to perform molecular docking between receptors and ligands in order to determine their binding affinity scores (kcal/mol) [12]. Let B*_mn_* denote the binding affinity score between *m*^th^ receptors (*m=*1, 2, …, q) and *n*^th^ ligands/agents (*n=*1, 2, ..., p). Receptors were sorted based on the descending order of their average scores $\left( \frac{1}{q}\sum_{n=1}^{p} B_{mn}, m=1,2\ldots q \right)$, while ligands/agents were ranked by the descending order of their average scores $\left( \frac{1}{p}\sum_{m=1}^{q} B_{\mathrm{mn}}, n=1,2,\ldots,p \right).$ This process was used to select the top-ranked ligands/agents as potential drug candidates. To evaluate the potential of our selected drug candidates, we conducted molecular docking analysis between each disease-causing protein and the selected drug candidates. We identified these key proteins through PPI analysis of cDEGs from three datasets for each of Mpox andT2D.

**Method S3. Molecular dynamics simulation**

Molecular Dynamics (MD) simulations were conducted to validate the results of molecular docking studies. MD simulations are crucial for assessing the stability of protein-ligand complexes, offering insights into fluctuations and conformational changes as the system reaches equilibrium [13]. In this study, MD simulations were used to analyze the stability of ligand-09, ligand-12, and standard binding complexes. Simulations were performed in a periodic water box for 100 nanoseconds using the CHARMM36 force field and Gromacs 2020 software [14,15]. The force field parameters for the ligands and proteins were generated with the CHARMM-GUI server [16]. The complexes were placed in a rectangular box with 10 Å buffer spacing, filled with TIP3P water molecules, and neutralized with salt and chloride ions. Energy minimization was carried out using the steepest descent method. Equilibration was performed at 310 K for 10 picoseconds using NVT (constant particle number, volume, and temperature) and NPT (constant particle number, pressure, and temperature) ensembles [17]. The Lincs method was used to constrain hydrogen bonds, with a 2 fs time step [18]. Van der Waals interactions were evaluated with a switching range of 12 to 14 Å and a cutoff of 14 Å. Electrostatic interactions were calculated with the Particle Mesh Ewald (PME) method, with a grid spacing of 1.2 Å [19]. After completing the 100 ns simulations, trajectory files were analyzed using Gromacs and VMD to study dynamic conformational changes and interactions within the complexes [20]. Metrics such as Root Mean Square Deviation (RMSD), Residue Root Mean Square Fluctuation (RMSF) and MM-GBSA binding free energy, were computed to assess the stability and behavior of the protein-ligand complexes throughout the simulation. The Schrödinger system constructor wizard was used to solvate the protein-ligand complex (PLC) with water molecules, employing the TIP3P solvent model [21,22]. The system was placed in an orthorhombic box (10 × 10 × 10 Å) with periodic boundary conditions, and a constant salt concentration of 0.15 M was maintained. After 100 ps of energy minimization, the resulting trajectory was used for molecular dynamics (MD) simulations. These simulations were performed for 100 nanoseconds (ns) with a time step of 2 femtoseconds (fs), and snapshots were recorded every 50 picoseconds (ps), resulting in a total of 5000 frames.The binding free energy (ΔGbind) of the protein-ligand interactions was calculated for each snapshot using the MM-GBSA method, implemented via the gmx_MM-PBSA tool. The binding free energy (Δ*G_bind_*) was calculated by using the following equation:

Δ*G_bind_* = *E_complex_* – (*E_protein_* + *E_ligand_*)

This comprehensive approach accounted for multiple interaction components, including van der Waals forces, electrostatic interactions, polar solvation effects, solvent-accessible surface area (SASA) contributions, and overall binding energies. Trajectories from the MD simulations conducted in explicit water (Desmond module) were converted to GROMACS-compatible formats (e.g., gro and .top files) using InterMol software [23–25].

**Supplementary Results**

**Results S1: Drug-likeness Properties**

After the molecular docking study, the four top-ranked drug molecules (Tecovirimat, Vindoline, Brincidofovir and Zanamivir) were investigated for drug-likeness prediction. The drug molecules exhibited superior pharmacokinetic properties with minimal violations of Lipinski’s Rule of Five (RO5). As per Lipinski’s RO5, these drug molecules follow almost all of the parameters except for the number of hydrogen bond acceptors (HBA) ≤ 10 and the number of hydrogen bond donors (HBD) ≤ 5 **(Table S13)**, and they successfully passed in the evaluation of Lipinski’s RO5. The drug molecules that showed better pharmacokinetics and followed all the rules of RO5 or at least two of the RO5 were considered drug-like compounds. Based on the RO5 violation and binding affinity score of the top-ranked drug molecules, the drug-likeness properties of the screened drug molecules are given in **Table S13**. Therefore, our predicted molecules satisfied most of the drug-likeness properties. Drug-like molecules should have a LogP ≤5 and a molecular weight ≤500 g/mol to ensure good membrane permeability and effective absorption [71]. All candidate drugs met these criteria, indicating they are suitably lipophilic and small for optimal drug-like properties. Under Veber’s rule, drug-like compounds should have a topological polar surface area (TPSA) ≤140 Å² and the number of rotatable bonds (ROTB) ROTB ≤10 [83]. All compounds except Zanamivir meet these criteria, with showing good bioavailability scores. All compounds, excluding Zanamivir, also align with Egan’s rule, supporting their potential for drug development.

**Supporting Tables**

| **Table S1**. Collection of drug agents for Monkeypox, smallpox and cHKGs Guided drugs agents | |
| --- | --- |
| **sources** | **Monkeypox** |
| [1] | Tecovirimat. |
| [2] | Tecovirimat , Brincidofovir . |
| [3] | Tecovirimat, brincidofovir and cidofovir. |
| [4] | Tecovirimat, cidofovir, brincidofovir, and trifluridine. |
|  |  |
| **Smallpox** | |
| [5] | TPOXX, Tembexa, Tecovirimat, Brincidofovir . |
| [6] | Tembexa. |
| **cHKGs-Guided drugs agents** | |
| [7] | Thyroglobulin, Amikacin, Lenograstim  Urokinase, Pembrolizumab, Ipilimumab  Nivolumab, Ruxolitinib, Terconazole  Totarol, Glycerol Phenylbutyrate, Cyclothiazide  4-Phenylbutyric Acid, Butoconazole, Hydroxyurea  Bortezomib, Halofantrine Hydrochloride, Sulconazole Nitrate  Vanillin, Eugenol, Ethyl Vanillin  Methyl Isobutyl Ketone, Benzaldehyde, Chloroquine  Sertaconazole Nitrate, Ciclopirox Olamine, Triclosan  Triclabendazole, Cinnamic Aldehyde, Tiopronin  Benzophenone, Sulisobenzone, Diacetylrhein  Iodoquinol, Thonzonium Bromide, Sodium Nitrite  Piperonyl Butoxide, Pipobroman, Buspirone Hydrochloride  Camphor Oil, Anthralin, Chloroxine  Fluconazole, Bifonazole, Pyrithione  Metronidazole, Oxybenzone, Amlexanox  Econazole Nitrate, Thiotepa, Citric Acid  Cariprazine, Physostigmine, Propylparaben  Dimercaprol, Pramipexole, Pentaerythritol Tetranitrate  Edaravone, Miconazole Nitrate, Carboplatin  Tetracycline, Clioquinol, 5-Hydroxytryptophan  Linoleic Acid, Ropinirole, Paclitaxel  Ifosfamide, Domperidone, Buspirone  Ketoconazole, Thimerosal, Dicumarol  Doconexent, Chloramphenicol, Dyclonine  Artemisinin, Oxymetholone, Oxytetracycline Hydrochloride  Rofecoxib, Cisplatin, Vilazodone Hydrochloride  Medroxyprogesterone Acetate, Mesoridazine, Rotigotine  Phenoxybenzamine Hydrochloride, Chlorprothixene, Liothyronine Sodium  Carbidopa Anhydrous, Daunorubicin Hydrochloride, Carmustine  Bifonazole, Methylergonovine, Phenoxybenzamine  Brimonidine, Clotrimazole, Catechin  Loxapine, Hexachlorophene, Chrysin  Cilostazol, Sulfinpyrazone. |
| **215 Antiviral agents** | |
| [8] | Vidarabine, Idoxuridine, Docosanol  Fomivirsen, Edoxudine, Trifluridine  Acyclovir, Sinecatechins, Inosine Pranobex  Tromantadine, Ganciclovir, Peginterferon Alfa-2a  Interferon Alfa-n1, Peginterferon Alfa-2b, Interferon Beta-1a  Interferon Alfacon-1, Enfuvirtide, Palivizumab  Oseltamivir, Nelfinavir, Indinavir  Nevirapine, Penciclovir, Tenofovir Disoproxil  Cidofovir, Famciclovir, Entecavir  Rimantadine, Zidovudine, Ritonavir  Foscarnet, Zanamivir, Valaciclovir  Efavirenz, Stavudine, Amprenavir  Delavirdine, Lamivudine, Adefovir Dipivoxil  Ribavirin, Emtricitabine, Didanosine  Amantadine, Tipranavir, Zalcitabine  Abacavir, Atazanavir, Saquinavir  Darunavir, Telbivudine, Fosamprenavir  Lopinavir, Valganciclovir, Maraviroc  Calanolide A, Pleconaril, Interferon Alfa  Leronlimab, Alovudine, Simeprevir  Etravirine, Peramivir, Clevudine  Raltegravir, Rilpivirine, Dolutegravir  Sofosbuvir, Ledipasvir, Ombitasvir  Paritaprevir, Tenofovir Alafenamide, Cenicriviroc  Pritelivir, Sorivudine, Vedroprevir  Beclabuvir, Atevirdine, Lobucavir  Racivir, Fiacitabine, Moroxydine  Metisazone, Adefovir, Podofilox  Ibacitabine, Tenofovir, Tecovirimat  Vesatolimod, Asunaprevir, Telaprevir  Maribavir, Boceprevir, Elvitegravir  Daclatasvir, Dasabuvir, Faldaprevir  Umifenovir, Letermovir, Baloxavir Marboxil  Carbovir, Ajoene, Pocapavir  HPA-23, Ammonium Trichlorotellurate, Aphidicolin  Dideoxyadenosine, Tilorone, Interferon Alfa-1b  Yimitasvir, Ulonivirine, Ensitrelvir  Semzuvolimab, Bersacapavir, Acemannan  Brivudine, Emivirine, Lapachone  Betulinic Acid, Elbasvir, Grazoprevir  Velpatasvir, Bictegravir, Voxilaprevir  Doravirine, Ibalizumab, Pibrentasvir  Glecaprevir, Alovudine F-18, Fialuridine  Rupintrivir, BMS-488043, UC-781  Elvucitabine, Taribavirin, Artesunate  Ruzasvir, Laninamivir Octanoate, Ingavirin  Dexelvucitabine, Laninamivir, Odalasvir  Adafosbuvir, Uprifosbuvir, Radavirsen  JE-2147, N-hydroxyguanidine, JPC-3210  Pirodavir, R-82913, Opaviraline  L-756423, Remdesivir, TMC-310911  Triazavirin, Valomaciclovir, Valomaciclovir Stearate  Ravidasvir, Islatravir, Baloxavir  GS-441524, Amenamevir, Favipiravir  Cabotegravir, Troclosene, Oxymatrine  Rintatolimod, Cilgavimab, Tixagevimab  Galidesivir, Brincidofovir, Netivudine  Ribavirin Monophosphate, Tenofovir Exalidex, Molnupiravir  Pradefovir Mesylate, Pradefovir, Interferon Alfa-2a  Interferon Alfa-2b, 2'-fluoro-5-ethylarabinosyluracil, Remestemcel-L  Ziresovir, Gimsilumab, Bamlanivimab  AZD7442, Atoltivimab, Maftivimab  Odesivimab, Gontivimab, Ansuvimab  Astegolimab, Zansecimab, Avdoralimab  Meplazumab, Aplaviroc, Capravirine  Dapivirine, Fostemsavir, PF-232798  Rovafovir Etalafenamide, Elsulfavirine, Tipranavir C-14  BMS-955176, Nirmatrelvir, Azodicarbonamide  Lenacapavir, Bulevirtide, Coblopasvir  Interferon Tau, Sotrovimab. |

| Table S2. Collection of type-2 diabetes (T2D) related candidate drug agents from published articles and other sources | |
| --- | --- |
| Articles | **Drug agents** |
| Bailey et al. 2016 [53] | Alogliptin, Saxagliptin, Sitagliptin, Lixisenatide, Empaglifl ozin, Liraglutide, Canaglifl ozin, Exenatide, Linagliptin, Dapaglifl ozin, Dulaglutide, Acarbose |
| Krentz et al. 2008 [54] | Sulfonylureas, Meglitinides, Metformin, Thiazolidinediones, α-Glucosidase, nhibitors, Insulin |
| Esposito et al. 2012 [55] | Basal, Biphasic, Prandial, Basal bolus, GLP-1 agonists, Exenatide LAR, DPP-4 inhibitors, AGI, Thiazolidinediones, Sulphonylureas, Glinides, Metformin |
| Moller et al. 2001 [56] | Insulin , Sulphonylureas, Metformin, Acarbose, Pioglitazone, rosiglitazone |
| Chehade et al. 2000 [57] | Acetohexamide, olbutamide, Chlorpropamide, Tolazamide, Glipizide, Glipizide, Glyburide, Glyburide micronised, Glimepiride, Gliquidone, Gliclazide |
| Deans et al. 2006 [58] | Statins, Fibrates, ACE inhibitors and ARBs, Aspirin, Metformin, Glitazones |
| Avery et al. 2008 [59] | Ciglitazone, Troglitazone, Pioglitazone, Englitazone, Darglitazone, Rosiglitazone, Isaglitazone |
| Paneni et al. 2017 [60] | Biguanides, Sulfonylureas, Thiazolidinediones, peptide-1 receptor, Dipeptidyl peptidase-4, Sodium glucose |
| Adeghate et al. 2021 [61] | Sulfonylureas, Biguanides, Inhibitors of α-glucosidase, Thiazolidinediones, Meglitinides, Dipeptidyl peptidase 4 inhibitors |
| Gupta et al. 2009 [62] | Insulin, Sulphonylurea, Biguanides, Acarbose , Thiazolidinediones , PPAR/ dual agonists, GLP-1 analogs |
| Tamborlane et al. 2016 [63] | Colesevelam, Exenatide, Linagliptin, Liraglutide, Saxagliptin, Sitagliptin, Taspoglutide, Empagliflozin, Exenatide, Alogliptin, Albiglutide, Omarigliptin, Dulaglutide, Lixisenatide, Sotagliflozin, Ertugliflozin |
| Lu et al. 2018 [64] | Insulin and its derivatives, Sulphonylurea, Biguanide, Acarbose, Voglibose, Pioglitazone, Exercise, Curcumin, Resveratol, α-thioctic acids, |
| Thrasher et al. 2017 [65] | Metformin, Albiglutide ,Dulaglutide, Exenatide, Exenatide XR, Liraglutide, Lixisenatide, Canagliflozin, Dapagliflozin, Empagliflozin, Alogliptin, Linagliptin, Sitagliptin, Saxagliptin, Glimepiride, Glipizide, Glyburide, Pioglitazone, Rosiglitazone |
| Kalsi et al 2015 [66] | Thiazolidinediones, Biguanide, Sulfonylureas, Meglitinides, Insulin |
| Olokoba et al. 2012 [67] | Biguanides, Sulfonylureas, Meglitinides, Thiazolidinediones, Alpha-Glucosidase Inhibitors, Incretin, Bromocriptine, Insulin |
| Kong et al. 2021 [68] | Berberine, Resveratrol , Emodin ,Ellagic acid, Epigallocatechin gallate, Curcumin, Baicalein, Naringenin, Hesperetin, Chrysin, Genistein, Kaempferol, Eriodictyol ,Apigenin ,Quercetin |
| Tripathi et al. 2013 [69] | Steroids, Triterpenoids, Saponins, Glycosides, Carbohydrates, Alkaloids, Flavonoids , Tannins & Phenolic, Proteins, Amino acid |
| Hassan et al. 2022 [70] | Tannin, Saponin, Alkaloid, Flavonoid, Phenol, Terpenoid, Carbohydrate |
| Yasmin et al. 2020 [71] | Singrin, Boeravinone E, Boeravinone D, Wedelolactone, Squamosamide , Taxifolin |
| Singh et al. 2019 [72] | Polyphenols, Amino Acid, Saponins, Terpenoids, Abscisic Acid, Lycopene and b-Carotene, Oxyphytosterol, Phytosterols/Stanols |
| González-Castejón et al. 2011 [73] | Epigallocatechin gallate, Cyandin and cyanidin 3-glucoside, Soy isoflavones mixture, Genistein, Naringin, Quercetin, Berberine, Resveratrol |
| Hariftyani et al. 2021 [74] | 6-Methoxykaempferol, α-Bisabolol, Anthecotulide, Apigenin, Apigenin-7-glucoside, Axillarin, Azulene, β-Sitosterol, Caffeic acid, Catechin, Chlorogenic acid, Chrysoeriol, Chrysosplenetin, Eupatoletin, Isoferulic acid, Isorhamnetin, Jaceidin, Kaempferol, Linoleic acid, Luteolin, Matricarin, Oleic acid, Patuletin, Quercetagetin-3,6,7,3',4'-, pentamethylether, Quercetin, Sinapic acid, Spinacetin, Stigmasterol, Thiamine, Umbeliferone. |
| Adefegha et al. 2013 [75] | saponins, alkaloids, terpenes, phenylpropanoids, isoprenoids, steroids, coumarins, flavonoids, phenolic acids, lignans, contain chemicals such as flavonoids, terpenoids, lignans, sulfides, polyphenolics, carotenoids, coumarins |
| Atere et al. 2017 [76] | (9Z,12Z)-octadeca-9,12-dienoic acid, 4-[Bis(2-hydroxyethyl)amino]phenyl]-1,1,2-ethylenetricarbonitrile, 1-(2-(methylthio)pyrimidin-4-yl)ethanone, 1-(2-chloropyridin-4-yl)ethanone, 1-(furan-2-yl)ethanone, 1,19-Eicosadiene, 2-methoxyphenol, 2-Pentadecanone, 2-Phenyloxazole-4-carboxylic acid, 2-Propenoic acid-3-(4-hydroxy-3-methethoxyphenly ferullic acid, 2,3-Dihydrobenzofuran-5-boronic acid, 2,6-Dimethoxyphenol, 7-decen-4-olide, 14-Methylpentadecanoic acid, Citronellyl isobutyrate, E stilbene, Inositol 1,4,5-trisphosphate, Methyl stearate, n-Hexadecanoic acid, Phenol, 2,6-bis(bromomethyl)-4-chloro-3,5-dimethyl, Phytol |
| Rampogu et al. 2018 [77] | Flavanoid, Tannin, Terpenoids, Alkaloids, Saponins |
| Kausar et al. 2021 [78] | 4,5-dimethyl-3-hydroxy-2(5H)-furanone, apigenin, bromelain, caffeic acid, cholecalciferol , dihydrokaempferol 7-o-glucopyranoside , galactomannan, genkwanin, isoimperatorin, luteolin, luteolin 7-o-glucoside, neohesperidin, oleanoic acid , pelargonidin-3-rutinoside, quercetin, quinic acid |
| Sari et al. 2021 [79] | 2,3-Butanediol, Glycerin, 4H-Pyran-4-one,2,3- dihydro-3,5-dihydroxy6-methyl, 5- Hydroxymethylfurfural, D-Glucose,6-O-à-Dgalactopyranosyl, Sucrose, Cyclooctasiloxane, hexadecamethyl -, n -Hexadecanoic acid, 4H -Pyran - 4 -one,5 - hydroxy - 2 – (hydroxymethyl) |
| Onaolapo et al. 2018 [80] | Flavonoids, Alkaloids, Glycosides, Polysaccharides, Terpenoids and steroids , Saponins Isothiocyanate, |
| Belobrajdic et al. 2013 [81] | Methionine, Cystine, Selenium, Folate, Choline, Tocopherols + tocotrienols, Carotenoids, Polyphenols, Phenolic acids, Phenolic acid, Ferulic acid, Flavanoids, Alkylresorcinols, Avenanthramides, Betaine, Phytosterols |
| Oboh et al. 2014[82] | Coumarin, p-Coumaric acid, o-Coumaric acid, Vanillic acid, Caffeic acid, Ferulic acid, Syringic acid, Sinapinic acid, Genistein, Apigenin, Naringenin, Kaempferol, Luteolin, Epicatechin, Epigallocatechin, Quercetin, 2-Phenylethyl-β-D-glucoside, Phenyl-6I-O-maloyl-β-D-glucoside |

| Table S3. List of HDEGs for each of Mpox and T2D |
| --- |
| HDEGs for Mpox |
| ABCB11, ABL1, ABO, ACAT1, ACO2, ACP1, ACTA2, ACTC1, ACTG2, ADAMTS13  ADCY2, ADD1, ADD2, ADH7, ADIPOQ, AFP, AGPAT5, AGPS, AIP, ALB, KIAA1551, LPAR2  ALDH8A1, ANGPT1, ANXA2P1, ANXA6, AOC3, AP2M1, APBA1, APOA1, APOC2, APOL1  AQP9, AR, ARAF, ARHGAP1, ARHGDIA, ARHGEF6, ARL2, ARSA, ASTN1, ATF5  ATG14, ATP6V0D1, ATP6V1A, ATRNL1, ATRX, AVPR1A, AZGP1, BCHE, BCKDHA, BCKDHB  BCO1, BFSP1, BMP8B, BMPR2, BMX, BRCA1, BST1, C8A, CA1, CA12  CACNA1C, CAMKK1, CAMKK2, CCR4, CCR8, CCRL2, CD160, CD19, CD40, CD40LG  CD5L, CD69, CDA, CDC25B, CDH1, CDK13, CDKN1C, CDKN2B, CDKN2C, CDX2  CEBPA, CENPE, CETN1, CETN2, CFL1, CHD2, CHRM1, CHRM2, CHRM3, CHRM4  CHRNA1, CHRNA3, CILP, CKM, CLCN4, CLIC3, CLTC, CNN1, CNN2, CNTRL  COL4A1, COL4A3, COPA, COQ2, COX7A1, CPA3, CPN2, CR1, CRH, CRYAA  CRYAB, CRYBA4, CSN3, CST1, CST8, CTLA4, CTNND1, CTSW, CYP7A1, DAG1  DCN, DDX24, DGKB, DGKI, DNAJB2, DNAJC5B, DPP3, DPYSL4, DSP, DSTYK  DTX4, EDF1, EDN2, EHHADH, EIF4B, EIF4H, ELF4, EN1, ENPP1, EPHA7  ERC2-IT1, ERLIN2, ERVW-1, EXOC3L4, EXOC5, EXTL3, EZR, F2, FABP3, FAM13A  FAM98A, FANCF, FCER2, FLRT3, FLVCR2, FOXO4, FRY, FSCN1, G6PD, GABRA1  GAS2L1, GCC2, GCLM, GEMIN5, GHR, GLP2R, GLRA2, GNLY, GOLGA4, GOSR1  GP2, GRID1, GSTA4, GTF3C3, HADHA, HERC3, HFE, HHIP, HLA-DOB, HLTF  HMGB3, HMMR, HNF4G, HOXA13, HOXA7, HOXC10, HOXC6, HOXD9, HSP90AA1, HSP90AB3P , HSPB1, HTR2B, HTR2C, ID2, IFNG, IGF1, IGF1R, IKZF1, IL2, IL21R , CAPRIN1, MRPS25, CIAO1, PHF23 ,IL22, IQGAP1, IQSEC1, KBTBD4, KCNA4, KCNAB1, KCNB1, KCNJ10, KCNK3, KCNMA1  KCNQ2, KCNQ4, KDM4A, KIAA0930, KIF20A, KIF22, KIF4A, KITLG, KLHDC3, KLK10  KLRG1, KRT14, KRT15, LALBA, LASP1, LCN1, LIMK2, LITAF, LNPEP, LPO  LST1, MAPK8IP3, MBL1P, MCM5, MEF2C, MEIS1, MEIS2, MFAP4, MKI67, MLLT1  MLLT3, MN1, MOBP, MPL, MPZL2, MTUS2, MUC12, MUC3A, MXD4, MYCN  MYH10, MYL2, MYO5A, NCAM2, NDUFA5, NEBL, NFE2L1, NFYC, NGB, NHS  NIPSNAP1, NKX2-1, NME6, NOS2, NPEPPS, NPY1R, NRAP, NTHL1, OASL, OLFML2A  PAEP, PAFAH2, PART1, PAX7, PBX1, PCDHB1, PCLO, PDCD6IP, PDE4B, PDGFRB  PDHA2, PDYN, PGRMC2, PLXDC1, POP1, PPL, PPP1R12B, PPP6C, PPT1, PRKAR1B  PRKAR2A, PRKCB, PRLR, PRPH2, PSAT1, PSG1, PSMB7, PSMC1, PSMD9, PTCH1  PTGIR, PTH1R, PTPA, PTPN21, PTPRB, PTPRC, RAF1, RALBP1, RBPJL, RDX  REG1A, RET, RHOBTB3, RIMS2, RIT2, RNF5, RNPEP, ROS1, RSL1D1, RYR1  SAG, SCAF11, SCARB1, SCN1A, SCN9A, SCRG1, SERPINA4, SERPINB13, SERPINE1, SETBP1  SGCD, SH2D1A, SH3BP5, SH3GL1, SIM2, SIRT4, SLC10A3, SLC12A3, SLC15A1, SLC15A2  SLC16A4, SLC22A3, SLC26A2, SLC2A5, SLC35A2, SLC35E3, SLC4A1, SLC5A4, SLC6A1, SLC6A7, SLC6A8, SLC6A9, SLC7A5, SLC8A1, SLC9A2, SLIT1, SMAD5-AS1, SMARCA2, SMARCC2, SNRPC , SNU13, SOX1, SOX10, SOX12, SOX5, SPAG11B, SPATA4, SPTAN1, SPTB, SPTBN1 , SPTBN2, SPTLC2, SRA1, SRMS, SRP68, SST, ST8SIA4, SUPT4H1, SUV39H1, SYCP2  SZT2, TAGLN, TARP, TBC1D9B, TBXA2R, TCF19, TCTA, TEF, TEX28, TGFB3  THRA, THSD7A, TIMP3, TNFRSF10C, TNFSF10, TNRC6B, TP63, TPGS2, TPM1, TRGV7  TRIM10, TRPM1, TSC22D3, TSPYL4, TYRP1, UGT2B4, USP49, USP6, VASH1, VCL  VIL1, VIPR1, VIPR2, WDR17, WDR19, WFS1, WNT2, WNT2B, WNT7B, WT1  XDH, XPC, ZADH2, ZAP70, ZBTB22, ZC3H3, ZC3H4, ZFY, ZNF10, ZNF17, PHF23, TLK2,  CDKL5, PRDM4, ZNF197, ZNF318, ABL1, ARHGEF17, BAZ1A, BBS5, BTG3, C2, CA2, CACNB4  CACNG3, CHST2, ELN, FAM84B, GRHL1, H19, HPX, ICAM1, IGLL5, KCNH4  KCNK5, KDM5D, LAMC2, LAMP3, LIFR, MAFF, MAP3K9, MLLT10, MYO1B, NEDD4L  NTF4, OCRL, OSBPL3, PAPPA, PAX4, PLAT, PLAU, PRTN3, RRH, SERPINA1, DMTN,  ST18, TDRKH, TEKT2, TGFA, TLE2, TNFAIP2, TUSC3, UBOX5, ZBTB21, ZMYND8  ZNRF4, ALDH8A1, ARID4B, ARRDC3, ASS1, ATP11 |
| HDEGs for T2D |
| AIP, ACRBP, ACP1, ACAT1, ACO2, ADD1, AKR7A2, ALDH1A1, ALDH4A1, ALDH8A1, ALDOC, ANP32A, ANXA6, ARF5, ARHGEF26, ARPP19, ASS1, ATF5, ATP6V1E1, B2M, BCKDHB, CABLES1, CAPN13, CAPRIN1, CBX1, CCDC8, CECR2, CERS4, CETN2, CH25H, CIAO1, CNST, CPD, CPT2, CPEB1, CTNNA3, DNAL1, EML2, ETFDH, ETV5, ETX1, FAM20B, FBXO45, FEM1B, FGB, FGD2, FKBP4, FPR1, GCLM, GLG1, GLIS3, GNL1, GORASP2, HADHA, HOMEZ, HSP90AA1, IGF1R, IGKC, JCHAIN, KBTBD4, KDM5D, KIF22, KLF16, LINC01116, LRPPRC, LSM14B, MACROD2, MBD1, MBP, MIA3, MCOLN3, MRPS25, MRPS30, MS4A6A, MTUS2, MYO1F, NAIF1, NECTIN3, NGRN, NIPSNAP1, NOP9, NPM3, NR3C2, OAZ2, ODC1, OTUD7B, PAFAH2, PAPPA, PBX1, PEBP1, PFKFB2, PFKFB3, PGRMC2, PITHD1, PCOLCE2, PDP2, PECP1, PHF23, PLAUR, POLR2C, PRDM4, PRKAR2A, PRKCH, PRPS1, PPP6C, PSMB7, PSMC1, PTPN3, RAB3GAP1, RAPH1, RCOR1, REL, RNF6, RSL1D1, SERPINA3, SLC2A1, SLC35A2, SLC35E3, SMIM10L1, SMIM13, SNU13, SNAPC3, SP1, SPTLC2, SRXN1, ST3GAL3, STX3, SYN1, TAPT1, TEF, TGFBR3, TLK2, TM9SF3, TMEM150C, TMEM37, TOMM70, TOP2A, TRAPPC11, TSSC4, TSHR, TTC7B, TUBGCP5, UBQLN1, UBE2Q1, UNC5D, USP30, VEGFA, VTI1B, WFS1, XPO4, YIF1B, ZNF134, ZNF318, ZNF428, ZNF654, ZNF770, LRPPRC, CABLES1, VEGFA. |

| Table S4. List of common HDEGs (cHDEGs) between Mpox and T2D |
| --- |
| ACAT1, ACP1, ACO2, ADD1, AIP, ALDH1A1, ALDH8A1, ANXA6, ASS1, ATF5, B2M, BCKDHB, CDK5R1, CETN2, ETV5, FGD2, FPR1, GCLM, GLIS3, HADHA, HSP90AA1, IGF1R, KBTBD4, KDM5D, KIF22, MTUS2, MS4A6A, MYO1F, NIPSNAP1, PAFAH2, PAPPA, PBX1, PFKFB2, PFKFB3, PGRMC2, PLAUR, PRKAR2A, PSMB7, PSMC1, PTGIR, P2RY1, PAPPA, PBX1, PGRMC2, PLAUR, PRKAR2A, PSMB7, PSMC1, PTGIR, RSL1D1, SLC35A2, SLC35E3, SNU13, SPTLC2, TEF, WFS1, ZNF318 |

| **Table S5.** Common 9 cHKGs out of top-ranked 15 cHKGs discovered by six different topological measures from PPI network based on STRING database. Scores of these common cHKGs with six different topological measure were given in columns 2-7. The ranks of cHKGs based on their scores were given in the last column. | | | | | | | |
| --- | --- | --- | --- | --- | --- | --- | --- |
| **chKGs** | Degree | Closeness | EPC | MNC | Betweenness | MCC | **Rank*** |
| HSP90AA1 | 26 | 36.66 | 679.89 | 25 | 679.893 | 41509 | **1** |
| ALDH1A1 | 22 | 32 | 237.21 | 24 | 237.2156 | 41422 | **2** |
| SNU13 | 20 | 31.5 | 234.70 | 21 | 234.7096 | 41340 | **3** |
| IGIF1R | 19 | 30.5 | 203.29 | 20 | 203.2902 | 41300 | **4** |
| B2M | 18 | 28.91 | 200.04 | 19 | 200.0455 | 41118 | **5** |
| ALDH8A1 | 17 | 28.80 | 171.20 | 17 | 171.2075 | 41074 | **6** |
| HADHA | 16 | 28.83 | 146.85 | 13 | 146.8569 | 40455 | **7** |
| ASS1 | 14 | 28.66 | 116.72 | 15 | 116.727 | 40370 | **8** |
| PLAUR | 12 | 28.41 | 103.31 | 11 | 103.3101 | 40344 | **9** |
| *HSP90AA1, ALDH1A1, ACO2, SNU13 and B2M secured ranks 1, 2, 3, 4, and 5, respectively, based on their scores across all measures. ALDH8A1 secured rank 6, supported by 5 measures, while HADHA secured rank 6, supported by 4 measures. Therefore, the ranks of HADHA, ASS1 & PLAUR were considered as 7, 8 and 9, respectively. | | | | | | | |

| **Table S6.** Common 9 cHKGs out of top-ranked 15 cHKGs discovered by six different topological measures from PPI network based on IMEx database. Scores of these common cHKGs with six different topological measure were given in columns 2-7. The ranks of cHKGs based on their scores were given in the last column as before. | | | | | | | |
| --- | --- | --- | --- | --- | --- | --- | --- |
| **cHKGs** | Degree | Closeness | EPC | MNC | Betweenness | MCC | **Rank**** |
| HSP90AA1 | 720 | 62.83 | 19 | 52 | 717283.5 | 770 | **1** |
| PSMB7 | 107 | 51.83 | 16.23 | 30 | 107375.82 | 122 | **2** |
| B2M | 79 | 50.66 | 16.20 | 28 | 62904.12 | 115 | **3** |
| ASS1 | 75 | 49.33 | 16.15 | 27 | 80341.5 | 101 | **4** |
| IGF1R | 65 | 50.33 | 14.77 | 20 | 55474.07 | 74 | **5** |
| HADHA | 58 | 46.16 | 14.35 | 52 | 49912.78 | 76 | **6** |
| UBC | 57 | 51.83 | 19 | 25 | 46127.27 | 57 | **7** |
| ALDH1A1 | 44 | 62.83 | 16.23 | 28 | 42950.48 | 45 | **8** |
| PRKAR2A | 40 | 50.66 | 16.20 | 30 | 30389.58 | 56 | **9** |
| **Rank was calculated as before in Table S5 | | | | | | | |

| **Table S7. Average r**ank of common 6 cHKGs between STRING and IMEx databases | | | |
| --- | --- | --- | --- |
| **cHKGs** | **Rank (R1) based on STRING database** | **Rank (R2) based on IMEx database** | **Average rank**  **(R1+R2)/2** |
| **HSP90AA1** | 1 | 1 | 1 |
| **B2M** | 5 | 3 | 4 |
| **IGF1R** | 4 | 5 | 4.5 |
| **ALDH1A1** | 2 | 8 | 5 |
| **ASS1** | 8 | 4 | 6 |
| **HADHA** | 7 | 6 | 6.5 |

| **Table S8.** The top significantly (adjusted *p*-value<0.05) enriched GO terms and KEGG pathways with cHKGs by Enrichr web tool | | | | |
| --- | --- | --- | --- | --- |
| **Biological Process (BP)** | | | | |
| **Annotation ID** | **Terms** | **Count** | **Adjusted *P-*Value** | **Accociated cHKGs** |
| GO:0071549 | cellular response to dexamethasone stimulus | **2** | 0.003349 | IGF1R, ASS1 |
| GO:0042026 | protein refolding | 2 | 0.0124091 | HSP90AA1, B2M |
| GO:0001934 | positive regulation of protein phosphorylation | 2 | 0.0127752 | HSP90AA1, ALDH1A1 |
| GO:0001889 | liver development | 2 | 0.0131550 | ASS1 |
| GO:0034756 | regulation of iron ion transport | 1 | 0.0131550 | B2M |
| GO:0071418 | cellular response to amine stimulus | 1 | 0.0131550 | ASS1 |
| **Molecular Function (MF)** | | | | |
| GO:0097718 | disordered domain specific binding | 1 | 0.003995 | HSP90AA1 |
| GO:0023026 | MHC class II protein complex binding | 2 | 0.003995 | HSP90AA1, B2M |
| GO:0030377 | urokinase plasminogen activator receptor activity | 1 | 0.004699 | ALDH1A1 |
| GO:0032564 | dATP binding | 1 | 0.004699 | HSP90AA1 |
| GO:0002135 | CTP binding | 1 | 0.004699 | HSP90AA1 |
| GO:0005009 | insulin receptor activity | 1 | 0.004973 | IGFR1 |
| **Cellular Component (CC)** | | | | |
| GO:0005925 | focal adhesion | 2 | 0.0007811 | ALDH1A1, B2M, HADHA |
| GO:0043025 | neuronal cell body | 3 | 0.0007811 | IGF1R, HSP90AA1, ASS1 |
| GO:0042470 | melanosome | 1 | 0.0005686 | HSP90AA1 |
| GO:0070062 | extracellular exosome | 4 | 0.000568 | HSP90AA1, B2M, ASS1, HADHA |
| GO:0005829 | cytosol | 4 | 0.0069836 | HSP90AA1, B2M, ASS1, HADHA |
| **KEGG Pathway** | | | | |
| hsa01230 | Biosynthesis of amino acids | 2 | 0.049658 | ASS1 |
| hsa04612 | Antigen processing and presentation | 2 | 0.039611 | HSP90AA1, B2M |
| hsa04914 | Progesterone-mediated oocyte maturation | 2 | 0.04135 | IGF1R, HSP90AA1 |

| **Table S9.** The top significantly (*p*-value<0.05) enriched GO terms and KEGG pathways with cHKGs by David web tool | | | |
| --- | --- | --- | --- |
| **Biological Process (BP)** | | | |
| **GO Term** | **Count** | ***P-*Value** | **Accociated cHKGs** |
| GO:0042026~protein refolding | 2 | 0.0106646 | HSP90AA1, B2M |
| GO:0071549~cellular response to dexamethasone stimulus | 2 | 0.0114568 | ASS1, IGF1R |
| GO:0045429~positive regulation of nitric oxide biosynthetic process | 2 | 0.0188003 | HSP90AA1, ASS1 |
| GO:0001889~liver development | 2 | 0.0344968 | ASS1 |
| GO:0071222~cellular response to lipopolysaccharide | 2 | 0.048742 | B2M, ASS1 |
| GO:0001934~positive regulation of protein phosphorylation | 2 | 0.0406587 | HSP90AA1, ALDH1A1 |
| **Molecular Function (MF)** | | | |
| GO:0042802~identical protein binding | 4 | 0.0066711 | HSP90AA1, B2M, ASS1, IGF1R |
| GO:0023026~MHC class II protein complex binding | 2 | 0.0111921 | HSP90AA1, B2M |
| GO:0097718~disordered domain specific binding | 1 | 0.0153095 | HSP90AA1 |
| GO:0003723~RNA binding | 2 | 0.0188031 | HSP90AA1, ASS1 |
| GO:0042803~protein homodimerization activity | 2 | 0.0367266 | HSP90AA1 |
| GO:0005198~structural molecule activity | 1 | 0.0476693 | B2M |
| **Cellular Component (CC)** | | | |
| GO:0070062~extracellular exosome | 5 | 3.74E-05 | HSP90AA1, HADHA, B2M, ASS1 |
| GO:0005925~focal adhesion | 3 | 4.67E-04 | ALDH1A1, B2M, HADHA |
| GO:0042470~melanosome | 2 | 6.89E-04 | HSP90AA1, |
| GO:0016020~membrane | 5 | 0.019125 | HSP90AA1, HADHA, ALDH1A1, B2M, IGF1R |
| **KEGG Pathway** | | | |
| hsa01230~Biosynthesis of amino acids | 2 | 0.0396585 | ASS1 |
| hsa04612~Antigen processing and presentation | 2 | 0.0396585 | HSP90AA1, B2M |
| hsa04914: Progesterone-mediated oocyte maturation | 2 | 0.0610709 | HSP90AA1, IGFR1 |

| **Table S10.** The top significantly ( *p*-value<0.05) enriched GO terms and KEGG pathways with cHKGs by GeneCodis web tool | | | |
| --- | --- | --- | --- |
| **Biological Process (BP)** | | | |
| **GO Term** | **Count** | ***P-*Value** | **Accociated cHKGs** |
| Negative Regulation of Intracellular Signal Transduction (GO:1902532) | **3** | 0.0000627 | HADHA; ALDH1A1; IGF1R |
| Positive Regulation of Nitric Oxide Biosynthetic Process (GO:0045429) | 2 | 0.0064464 | HSP90AA1; ASS1 |
| Positive Regulation of Nitric Oxide Metabolic Process (GO:1904407) | 2 | 0.0064464 | HSP90AA1; ASS1 |
| Regulation of Nitric Oxide Biosynthetic Process (GO:0045428) | 2 | 0.0064464 | HSP90AA1; ASS1 |
| Positive Regulation of Binding (GO:0051099) | 2 | 0.0281564 | ALDH1A1; B2M |
| **Molecular Function (MF)** | | | |
| Disordered Domain Specific Binding (GO:0097718) | 2 | 0.0111921 | HSP90AA1; |
| MHC Class II Protein Complex Binding (GO:0023026) | 1 | 0.0153095 | HSP90AA1; B2M |
| Protein Homodimerization Activity (GO:0042803) | 2 | 0.0188031 | HSP90AA1; B2M |
| cAMP Binding (GO:0030552) | 1 | 0.0255933 | HADHA |
| Insulin Receptor Binding (GO:0005158) | 1 | 0.0312654 | IGFR1 |
| **Cellular Component (CC)** | | | |
| Focal Adhesion (GO:0005925) | 3 | 5.67E-04 | HADHA; ALDH1A1; B2M |
| Cell-Substrate Junction (GO:0030055) | 3 | 5.67E-04 | HADHA; ALDH1A1; B2M |
| Intracellular Organelle Lumen (GO:0070013) | 4 | 0.0045927 | HSP90AA1; ALDH1A1; B2M |
| **KEGG Pathway** | | | |
| Antigen processing and presentation | 2 | 0.0212418 | HSP90AA1; B2M |
| Fluid shear stress and atherosclerosis | 2 | 0.0334414 | HSP90AA1; ASS1 |
| Arginine biosynthesis | 1 | 0.0444788 | ASS1 |
| Progesterone-mediated oocyte maturation | 2 | 0.0461864 | HSP90AA1; IGF1R |

| **Table S11:** Docking/(binding affinity) scores (kcal/mol) between the proposed target genes/proteins (receptors) and top ordered 40 candidate drugs Out of 483) | | | | | | | | |
| --- | --- | --- | --- | --- | --- | --- | --- | --- |
| **Drug** | **ASS1** | **ALDH1A1** | **B2M** | **IGF1R** | **RELA** | **HADHA** | **HSP90AA1** | **YY1** |
| **Tecovirimat** | -12.2 | -10.2 | -12.3 | -11.5 | -11.6 | -11.2 | -9.5 | -8.5 |
| **Vindoline** | -12.8 | -9.6 | -11.2 | -10.4 | -10.6 | -10.4 | -9 | -8.3 |
| **Brincidofovir** | -12.6 | -7.6 | -11 | -10.9 | -10.3 | -10.5 | -10.9 | -8.5 |
| **Zanamivir** | -12 | -9.3 | -11.9 | -9.5 | -7.4 | -8.8 | -9.2 | -8.1 |
| **Herbacetin** | -11.9 | -9.9 | -10.3 | -9.9 | -9 | -7.8 | -7.5 | -7.2 |
| **Pleconaril** | -11.7 | -8.5 | -9 | -7.3 | -8.1 | -7.3 | -7.1 | -7.1 |
| **Silymarin** | -11.2 | -8.1 | -10.4 | -7.9 | -7.4 | -7.7 | -7.4 | -7.2 |
| **Tolazamide** | -11 | -8.8 | -10.4 | -7.9 | -7.2 | -7.2 | -8.1 | -6.8 |
| **Glycosides** | -11 | -10.6 | -8.4 | -7.5 | -7.9 | -7.1 | -6.9 | -6.5 |
| **Delavirdine** | -10.8 | -9.6 | -9.1 | -8 | -8.7 | -7.2 | -6.8 | -6.2 |
| **Beclabuvir** | -10.8 | -8.4 | -10 | -7.5 | -7.8 | -7.2 | -7.2 | -6.7 |
| **Acacetin** | -10.7 | -9.7 | -10.2 | -7.9 | -7.5 | -7.5 | -7 | -6.8 |
| **Gossypetin** | -10.6 | -8.8 | -10.5 | -7.9 | -6.7 | -6.9 | -6.8 | -6 |
| **Campesterol** | -10.5 | -8.1 | -9.3 | -7.8 | -7 | -7.9 | -6.8 | -6.8 |
| **Laninamivir** | -10.5 | -9.6 | -8.7 | -7.2 | -6.4 | -7.4 | -6.5 | -6 |
| **Sorbifolin** | -10.4 | -9.7 | -9.3 | -7.8 | -7.9 | -7.3 | -7.4 | -6.8 |
| **Glyburide** | -10.3 | -8.7 | -9.3 | -7.8 | -7.3 | -7.2 | -6.8 | -6.7 |
| **Carbovir** | -10.1 | -9.7 | -10 | -8.2 | -7.3 | -7.5 | -7.3 | -6.5 |
| **Doravirine** | -10.1 | -10 | -9.2 | -7.5 | -8.2 | -6.8 | -7.4 | -6.8 |
| **Aspalathin** | -10.1 | -10.1 | -9.1 | -6.3 | -7.2 | -7.6 | -6.8 | -6.6 |
| **Gossypol** | -10.1 | -8.6 | -9.3 | -7.4 | -7.2 | -7.2 | -7.2 | -6.8 |
| **Tecovirimat** | -10.1 | -9.8 | -9.2 | -7.5 | -7 | -7.2 | -7.2 | -6.7 |
| **Kolaviron** | -10 | -7.4 | -9.1 | -7.8 | -6.2 | -6.5 | -6.2 | -6.7 |
| **Bergenin** | -9.9 | -9 | -10 | -7.5 | -8.2 | -6.8 | -7.4 | -6.8 |
| **Tenofovir** | -9.9 | -7.1 | -10.1 | -7.2 | -7.2 | -7.2 | -7.2 | -6.8 |
| **Canagliflozin** | -9.9 | -9.1 | -10.2 | -7.1 | -7.9 | -6.9 | -6.7 | -6.9 |
| **Ibacitabine** | -9.9 | -10.6 | -8.8 | -7 | -7.1 | -7.2 | -6.7 | -6.7 |
| **Boceprevir** | -9.9 | -8.1 | -8.9 | -7.5 | -6.7 | -7.1 | -6.9 | -6.2 |
| **Grazoprevir** | -9.9 | -9.8 | -8.4 | -6.5 | -6.2 | -7.6 | -7 | -6.6 |
| **Polyphenols** | -9.9 | -8.4 | -9.8 | -7.4 | -7.9 | -7.3 | -6.7 | -6.4 |
| **Berberine** | -9.9 | -10.6 | -8.7 | -6.6 | -6.8 | -6.9 | -6.3 | -6.4 |
| **Resveratrol** | -9.8 | -7.3 | -10 | -7.4 | -8 | -6.9 | -6.8 | -6.5 |
| **Podofilox** | -9.8 | -10.3 | -9.4 | -7.2 | -6.5 | -6.3 | -7.6 | -6.3 |
| **Ulonivirine** | -9.8 | -8.5 | -10.2 | -6.9 | -7.3 | -6.9 | -6.6 | -6 |
| **Sofosbuvir** | -9.8 | -8.2 | -8.2 | -6.8 | -7.3 | -6.8 | -6 | -6.1 |
| **Baloxavir marboxil** | -9.8 | -9 | -9.7 | -7.5 | -6.2 | -7 | -6.4 | -6.8 |
| **Salvigenin** | -9.8 | -8.5 | -9.1 | -7.1 | -6.5 | -6.7 | -7 | -6.5 |
| **Baptigenin** | -9.8 | -9 | -9 | -7.2 | -6.9 | -7.2 | -6.1 | -6.7 |
| **Polysaccharides** | -9.8 | -9.3 | -9.6 | -6 | -6 | -7.8 | -5.9 | -5.9 |
| **Sorivudine** | -9.8 | -9.7 | -9.4 | -7.9 | -6.6 | -7 | -6 | -4.7 |

| **Table S12:** Docking/(binding affinity) scores (kcal/mol) between the individual disease-causing proteins (receptors) and top ordered 4 candidate drugs (Out of 483) | | | | | | | | | | |
| --- | --- | --- | --- | --- | --- | --- | --- | --- | --- | --- |
| **Drug** | **CDH1** | **PTPRC** | **ALDH4A1** | **PDGFRB** | **ACAT1** | **BCKDHB** | **UBE3C** | **RDX** | **HSPA9** | **LRPPRC** |
| **Tecovirimat** | -11.8 | -10.4 | -10.8 | -10.5 | -10.3 | -9.9 | -9.5 | -9 | -8.2 | -8.1 |
| **Vindoline** | -11.4 | -10.9 | -10.7 | -9.8 | -8.6 | -8.4 | -8.7 | -8.5 | -8 | -7.9 |
| **Brincidofovir** | -11 | -9.3 | -10.1 | -10.4 | -8.5 | -8.8 | -8.3 | -8.8 | -8.3 | -7.5 |
| **Zanamivir** | -8.6 | -7.7 | -7.5 | -7.7 | -7.7 | -7.9 | -7.6 | -7.4 | -7.3 | -7.3 |

**Table S13:** Drug likeness & pharmacokinetics properties of top ranked 4 candidate drug molecules

| Compounds | Molecular weight  (MW) | Log P | HBA | HBD | TPSA  (Å²) | Rotatable bond  (ROTB) | Bioavailability  Score | Lipinski’s Rule | Veber Rule | Egan |
| --- | --- | --- | --- | --- | --- | --- | --- | --- | --- | --- |
|  | SB≤500 | SB≤5 | SB≤10 | SB≤5 | SB≤140 | SB≤5 |  |  |  |  |
| Tecovirimat | 376.33 | 2.61 | 6 | 1 | 66.48 | 4 | 0.55 | 0 | 0 | 0 |
| Brincidofovir | 561.69 | 4.89 | 8 | 3 | 155.94 | 26 | 0.55 | 1 | 2 | 1 |
| Vindoline | 456.53 | 1.5 | 7 | 1 | 88.54 | 7 | 0.55 | 0 | 0 | 0 |
| Zanamivir | 332.31 | -3.19 | 8 | 7 | 200.72 | 7 | 0.17 | 1 | 1 | 1 |

N.B: HBA= Hydrogen bond acceptor, HBD=Hydrogen bond donor, SB= Should Be, and, TPSA= Topological Polar Surface Area

**Supplementary Figures**

**
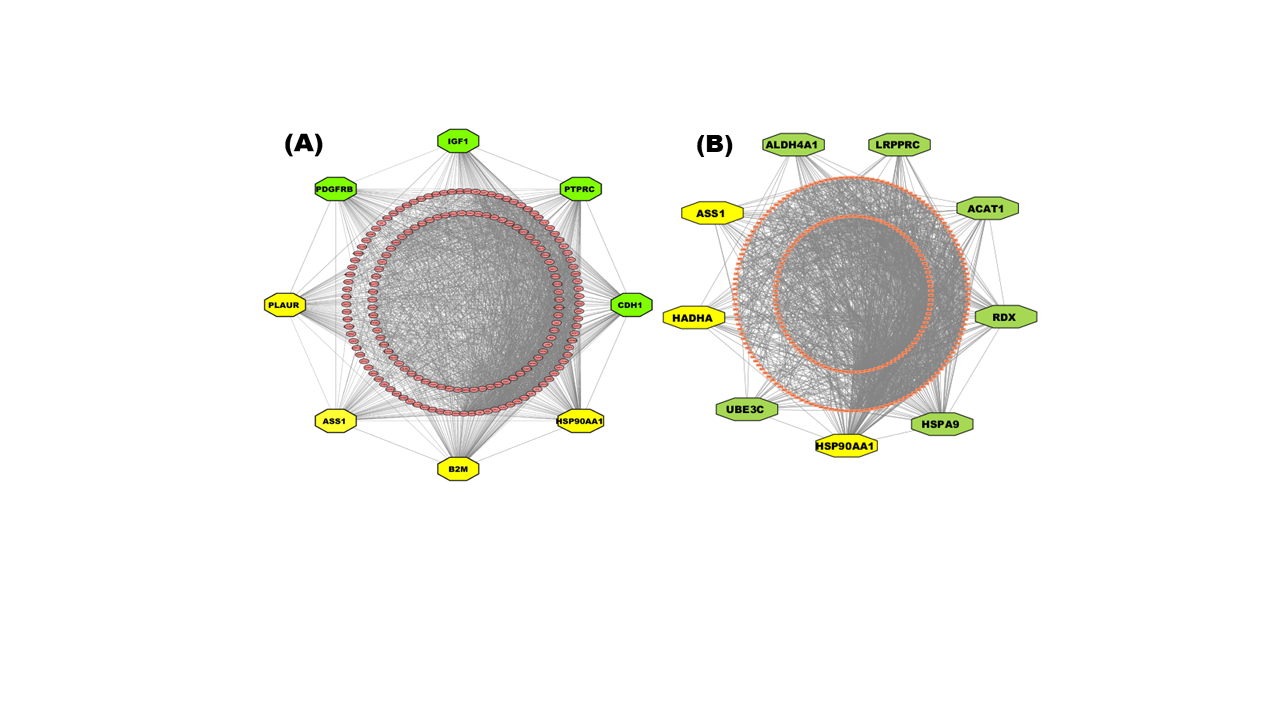
**

**Figure S1:** (A) Protein-protein interactions (PPIs) of HDEGs for Mpox are shown with green and yellow octagonal nodes. Yellow nodes represent proteins common with our proposed receptors, while green nodes indicate unique proteins. (B) Protein-protein interactions (PPIs) of HDEGs for T2D are shown with green and yellow octagonal nodes. Yellow nodes represent proteins common with our proposed receptors, while green nodes indicate unique proteins.

**References:**

1. Smyth, G.K. Linear Models and Empirical Bayes Methods for Assessing Differential Expression in Microarray Experiments. *Stat. Appl. Genet. Mol. Biol.* **2004**, *3*, doi:10.2202/1544-6115.1027.

2. Ritchie, M.E.; Phipson, B.; Wu, D.; Hu, Y.; Law, C.W.; Shi, W.; Smyth, G.K. Limma Powers Differential Expression Analyses for RNA-Sequencing and Microarray Studies. *Nucleic Acids Res.* **2015**, *43*, e47, doi:10.1093/nar/gkv007.

3. Reza, M.S.; Hossen, M.A.; Harun-Or-Roshid, M.; Siddika, M.A.; Kabir, M.H.; Mollah, M.N.H. Metadata Analysis to Explore Hub of the Hub-Genes Highlighting Their Functions, Pathways and Regulators for Cervical Cancer Diagnosis and Therapies. *Discov. Oncol.* **2022**, *13*, doi:10.1007/s12672-022-00546-6.

4. Kabir, S.R.; Islam, T.; Mollah, M.N.H. 2,4-Dipropylphloroglucinol Inhibits the Growth of Human Lung and Colorectal Cancer Cells through Induction of Apoptosis. *Med. Oncol.* **2023**, *40*, 1–12, doi:10.1007/s12032-023-01986-y.

5. Saur, I.M.L.; Panstruga, R.; Schulze-Lefert, P. NOD-like Receptor-Mediated Plant Immunity: From Structure to Cell Death. *Nat. Rev. Immunol.* **2021**, *21*, 305–318, doi:10.1038/s41577-020-00473-z.

6. Varadi, M.; Anyango, S.; Deshpande, M.; Nair, S.; Natassia, C.; Yordanova, G.; Yuan, D.; Stroe, O.; Wood, G.; Laydon, A.; et al. AlphaFold Protein Structure Database: Massively Expanding the Structural Coverage of Protein-Sequence Space with High-Accuracy Models. *Nucleic Acids Res.* **2022**, *50*, D439–D444, doi:10.1093/nar/gkab1061.

7. Schwede, T.; Kopp, J.; Guex, N.; Peitsch, M.C. SWISS-MODEL: An Automated Protein Homology-Modeling Server. *Nucleic Acids Res.* **2003**, *31*, 3381–3385, doi:10.1093/nar/gkg520.

8. Kim, S.; Chen, J.; Cheng, T.; Gindulyte, A.; He, J.; He, S.; Li, Q.; Shoemaker, B.A.; Thiessen, P.A.; Yu, B.; et al. PubChem 2019 Update: Improved Access to Chemical Data. *Nucleic Acids Res.* **2019**, *47*, D1102–D1109, doi:10.1093/nar/gky1033.

9. D.studio Discovery Studio Visualizer. *Discovery* **2014**, *pp.3*-*5*.

10. Morris, G.M.; Ruth, H.; Lindstrom, W.; Sanner, M.F.; Belew, R.K.; Goodsell, D.S.; Olson, A.J. Software News and Updates AutoDock4 and AutoDockTools4: Automated Docking with Selective Receptor Flexibility. *J. Comput. Chem.* **2009**, *30*, 2785–2791, doi:10.1002/jcc.21256.

11. Hanwell, M.D.; Curtis, D.E.; Lonie, D.C.; Vandermeersch, T.; Zurek, E.; Hutchison, G.R. 1758-2946-4-17. *J. Cheminform.* **2012**, *4*, 1–17.

12. Trott, O.; Olson, A.J. AutoDock Vina: Improving the Speed and Accuracy of Docking with a New Scoring Function, Efficient Optimization, and Multithreading. *J. Comput. Chem.* **2009**, *31*, NA-NA, doi:10.1002/jcc.21334.

13. Kaya, G.; Noma, S.A.A.; Barut Celepci, D.; Bayıl, İ.; Taskin-Tok, T.; Gök, Y.; Ateş, B.; Aktaş, A.; Aygün, M.; Tezcan, B. Design, Synthesis, Spectroscopic Characterizations, Single Crystal X-Ray Analysis, in Vitro Xanthine Oxidase and Acetylcholinesterase Inhibitory Evaluation as Well as in Silico Evaluation of Selenium-Based N-Heterocyclic Carbene Compounds. *J. Biomol. Struct. Dyn.* **2023**, *41*, 11728–11747, doi:10.1080/07391102.2022.2163696.

14. Huang, J.; MacKerell, A.D.J. CHARMM36 All-Atom Additive Protein Force Field: Validation Based on Comparison to NMR Data. *J. Comput. Chem.* **2013**, *34*, 2135–2145, doi:10.1002/jcc.23354.

15. Bekker, H.; Berendsen, H.J.C.; Van Der Spoel, D. Gromacs: A Parallel Computer for Molecular Dynamics Simulations Design of Polarizable Analytical Potentials View Project Density Matrix Evolution View Project. *Phys. Comput.* **1993**, *92*, 252–256.

16. Brooks, B.R.; Brooks, C.L. 3rd; Mackerell, A.D.J.; Nilsson, L.; Petrella, R.J.; Roux, B.; Won, Y.; Archontis, G.; Bartels, C.; Boresch, S.; et al. CHARMM: The Biomolecular Simulation Program. *J. Comput. Chem.* **2009**, *30*, 1545–1614, doi:10.1002/jcc.21287.

17. Akash, S.; Islam, M.R.; Bhuiyan, A.A.; Islam, M.N.; Bayıl, I.; Saleem, R.M.; Albadrani, G.M.; Al-Ghadi, M.Q.; Abdel-Daim, M.M. In Silico Evaluation of Anti-Colorectal Cancer Inhibitors by Resveratrol Derivatives Targeting Armadillo Repeats Domain of APC: Molecular Docking and Molecular Dynamics Simulation. *Front. Oncol.* **2024**, *14*, 1–15, doi:10.3389/fonc.2024.1360745.

18. Hess, B.; Bekker, H.; Berendsen, H.J.C.; Fraaije, J.G.E.M. LINCS: A Linear Constraint Solver for Molecular Simulations. *J. Comput. Chem.* **1997**, *18*, 1463–1472, doi:10.1002/(SICI)1096-987X(199709)18:12<1463::AID-JCC4>3.0.CO;2-H.

19. George, A.; Mondal, S.; Purnaprajna, M.; Athri, P. Review of Electrostatic Force Calculation Methods and Their Acceleration in Molecular Dynamics Packages Using Graphics Processors. *ACS omega* **2022**, *7*, 32877–32896, doi:10.1021/acsomega.2c03189.

20. Humphrey, W.; Dalke, A.; Schulten, K. VMD: Visual Molecular Dynamics. *J. Mol. Graph.* **1996**, *14*, 27-28,33-38, doi:10.1016/0263-7855(96)00018-5.

21. Arya, P.K.; Mandal, P.; Barik, K.; Singh, D.V.; Kumar, A. Computational Evaluation of Phytochemicals Targeting DNA Topoisomerase I in Leishmania Donovani: Molecular Docking and Molecular Dynamics Simulation Studies. *J. Biomol. Struct. Dyn.* 1–14, doi:10.1080/07391102.2023.2256865.

22. Nutt, D.R.; Smith, J.C. Molecular Dynamics Simulations of Proteins:  Can the Explicit Water Model Be Varied? *J. Chem. Theory Comput.* **2007**, *3*, 1550–1560, doi:10.1021/ct700053u.

23. Valdés-Tresanco, M.S.; Valdés-Tresanco, M.E.; Valiente, P.A.; Moreno, E. Gmx_MMPBSA: A New Tool to Perform End-State Free Energy Calculations with GROMACS. *J. Chem. Theory Comput.* **2021**, *17*, 6281–6291, doi:10.1021/acs.jctc.1c00645.

24. Miller, B.R.I.I.I.; McGee, T.D.J.; Swails, J.M.; Homeyer, N.; Gohlke, H.; Roitberg, A.E. MMPBSA.Py: An Efficient Program for End-State Free Energy Calculations. *J. Chem. Theory Comput.* **2012**, *8*, 3314–3321, doi:10.1021/ct300418h.

25. Shirts, M.R.; Klein, C.; Swails, J.M.; Yin, J.; Gilson, M.K.; Mobley, D.L.; Case, D.A.; Zhong, E.D. Erratum to: Lessons Learned from Comparing Molecular Dynamics Engines on the SAMPL5 Dataset (Journal of Computer-Aided Molecular Design, (2017), 31, 1, (147-161), 10.1007/S10822-016-9977-1). *J. Comput. Aided. Mol. Des.* **2017**, *31*, 777, doi:10.1007/s10822-017-0043-4.

26. Li, W.; Meng, X.; Yuan, H.; Xiao, W.; Zhang, X. A Novel Immune-Related CeRNA Network and Relative Potential Therapeutic Drug Prediction in CcRCC. *Front. Genet.* **2022**, *12*, 1–13, doi:10.3389/fgene.2021.755706.

27. Lu, M.; Xiao, L.; Xu, B.; Gao, Q. Identification of Novel Genes and Associated Drugs in AdvanceClear Cell Renal Cell Carcinoma by Bioinformatic Methods. *Tohoku J. Exp. Med.* **2022**, *258*, 79–90, doi:10.1620/tjem.2022.J059.

28. Sun, M.; Lughezzani, G.; Perrotte, P.; Karakiewicz, P.I. Treatment of Metastatic Renal Cell Carcinoma. *Nat. Rev. Urol.* **2010**, *7*, 327–338, doi:10.1038/nrurol.2010.57.

29. Xiao, G.F.; Yan, X.; Chen, Z.; Zhang, R.J.; Liu, T.Z.; Hu, W.L. Identification of a Novel Immune-Related Prognostic Biomarker and Small-Molecule Drugs in Clear Cell Renal Cell Carcinoma (CcRCC) by a Merged Microarray-Acquired Dataset and TCGA Database. *Front. Genet.* **2020**, *11*, 1–14, doi:10.3389/fgene.2020.00810.

30. Kramer, M.W.; Merseburger, A.S.; Peters, I.; Waalkes, S.; Kuczyk, M.A. Systemische Und Operative Therapie Des Metastasierten Nierenzellkarzinoms. *Urol. - Ausgabe A* **2012**, *51*, 217–225, doi:10.1007/s00120-011-2713-5.

31. Bai, S.; Wu, Y.Y.; Yan, Y.; Shao, S.; Zhang, J.; Liu, J.; Hui, B.; Liu, R.; Ma, H.; Zhang, X.; et al. Construct a CircRNA/MiRNA/MRNA Regulatory Network to Explore Potential Pathogenesis and Therapy Options of Clear Cell Renal Cell Carcinoma. *Sci. Rep.* **2020**, *10*, 1–15, doi:10.1038/s41598-020-70484-2.

32. Jonasch, E.; Atkins, M.B.; Chowdhury, S.; Mainwaring, P. Combination of Anti-Angiogenics and Checkpoint Inhibitors for Renal Cell Carcinoma: Is the Whole Greater Than the Sum of Its Parts? *Cancers (Basel).* **2022**, *14*, doi:10.3390/cancers14030644.

33. Jiang, A.; Song, J.; Fang, X.; Fang, Y.; Wang, Z.; Liu, B.; Wu, Z.; Qu, L.; Luo, P.; Wang, L. A Novel Thinking: DDR Axis Refines the Classification of CcRCC with Distinctive Prognosis, Multi Omics Landscape and Management Strategy. *Front. Public Heal.* **2022**, *10*, doi:10.3389/fpubh.2022.1029509.

34. DRUGBANK online Advanced Kidney Cancer-DRUGBANK Online.

35. NIH NIH The Cancer Genome Atlas Program - NCI. *Cancer Genome Atlas Progr.*

36. Drugs.com Drugs Used to Treat Renal Cell Carcinoma-Drugs.Com. *Drugs used to treat Ren. Cell Carcinoma*.

37. Hossen, M.B.; Islam, M.A.; Reza, M.S.; Kibria, M.K.; Horaira, M.A.; Tuly, K.F.; Faruqe, M.O.; Kabir, F.; Mollah, M.N.H. Robust Identification of Common Genomic Biomarkers from Multiple Gene Expression Profiles for the Prognosis, Diagnosis, and Therapies of Pancreatic Cancer. *Comput. Biol. Med.* **2023**, *152*, 106411, doi:10.1016/j.compbiomed.2022.106411.

38. Gao, Y.; Chen, S.; Sun, J.; Su, S.; Yang, D.; Xiang, L.; Meng, X. Traditional Chinese Medicine May Be Further Explored as Candidate Drugs for Pancreatic Cancer: A Review. *Phyther. Res.* **2021**, *35*, 603–628, doi:10.1002/ptr.6847.

39. Ilmer, M.; Westphalen, C.B.; Niess, H.; D’Haese, J.G.; Angele, M.K.; Werner, J.; Renz, B.W. Repurposed Drugs in Pancreatic Ductal Adenocarcinoma: An Update. *Cancer J. (United States)* **2019**, *25*, 134–138, doi:10.1097/PPO.0000000000000372.

40. Sugarman, R.; Patel, R.; Sharma, S.; Plenker, D.; Tuveson, D.; Saif, M.W. Pharmacokinetics and Pharmacodynamics of New Drugs for Pancreatic Cancer. *Expert Opin. Drug Metab. Toxicol.* 2019, *15*.

41. Gao, G.; Liu, C.; Jain, S.; Li, D.; Wang, H.; Zhao, Y.; Liu, J. Potential Use of Aptamers for Diagnosis and Treatment of Pancreatic Cancer. *J. Drug Target.* **2019**, *27*, 853–865, doi:10.1080/1061186X.2018.1564924.

42. Ma, Y.; Hu, J.; Zhang, N.; Dong, X.; Li, Y.; Yang, B.; Tian, W.; Wang, X. Prediction of Candidate Drugs for Treating Pancreatic Cancer by Using a Combined Approach. *PLoS One* **2016**, *11*, 1–13, doi:10.1371/journal.pone.0149896.

43. Assaf, E.; Verlinde-Carvalho, M.; Delbaldo, C.; Grenier, J.; Sellam, Z.; Pouessel, D.; Bouaita, L.; Baumgaertner, I.; Sobhani, I.; Tayar, C.; et al. 5-Fluorouracil/Leucovorin Combined with Irinotecan and Oxaliplatin (FOLFIRINOX) as Second-Line Chemotherapy in Patients with Metastatic Pancreatic Adenocarcinoma. *Oncology* **2011**, *80*, 301–306, doi:10.1159/000329803.

44. Khare, V.; Alam, N.; Saneja, A.; Dubey, R.D.; Gupta, P.N. Targeted Drug Delivery Systems for Pancreatic Cancer. *J. Biomed. Nanotechnol.* **2014**, *10*, 3462–3482, doi:10.1166/jbn.2014.2036.

45. Luo, Y.; Hu, J.; Liu, Y.; Li, L.; Li, Y.; Sun, B.; Kong, R. Invadopodia: A Potential Target for Pancreatic Cancer Therapy. *Crit. Rev. Oncol. Hematol.* **2021**, *159*, doi:10.1016/j.critrevonc.2021.103236.

46. Yarchoan, M.; Myzak, M.C.; Johnson, B.A.; De Jesus-Acosta, A.D.; Le, D.T.; Jaffee, E.M.; Azad, N.S.; Donehower, R.C.; Zheng, L.; Oberstein, P.E.; et al. Olaparib in Combination with Irinotecan, Cisplatin, and Mitomycin c in Patients with Advanced Pancreatic Cancer. *Oncotarget* **2017**, *8*, doi:10.18632/oncotarget.17237.

47. Vaishampayan, U.N. An Evaluation of Olaparib for the Treatment of Pancreatic Cancer. *Expert Opin. Pharmacother.* 2021, *22*.

48. Zheng, Y.; Wu, C.; Yang, J.; Zhao, Y.; Jia, H.; Xue, M.; Xu, D.; Yang, F.; Fu, D.; Wang, C.; et al. Insulin-like Growth Factor 1-Induced Enolase 2 Deacetylation by HDAC3 Promotes Metastasis of Pancreatic Cancer. *Signal Transduct. Target. Ther.* **2020**, *5*, doi:10.1038/s41392-020-0146-6.

49. El Hassouni, B.; Mantini, G.; Immordino, B.; Peters, G.J.; Giovannetti, E. CX-5461 Inhibits Pancreatic Ductal Adenocarcinoma Cell Growth, Migration and Induces DNA Damage. *Molecules* **2019**, *24*, doi:10.3390/molecules24244445.

50. MarElia, C.B.; Sharp, A.E.; Shemwell, T.A.; Clare Zhang, Y.; Burkhardt, B.R. Anemarrhena Asphodeloides Bunge and Its Constituent Timosaponin-AIII Induce Cell Cycle Arrest and Apoptosis in Pancreatic Cancer Cells. *FEBS Open Bio* **2018**, *8*, 1155–1166, doi:10.1002/2211-5463.12457.

51. National Cancer Institute Drugs Approved for Pancreatic Cancer - National Cancer Institute.

52. Gu, H.; Yuan, G. Identification of Potential Key Genes for SARS-CoV-2 Infected Human Bronchial Organoids Based on Bioinformatics Analysis. *bioRxiv* **2020**, 2020.08.18.256735, doi:10.1101/2020.08.18.256735.

53. Bailey, C.J.; Tahrani, A.A.; Barnett, A.H. Future Glucose-Lowering Drugs for Type 2 Diabetes. *Lancet Diabetes Endocrinol.* **2016**, *4*, 350–359, doi:10.1016/S2213-8587(15)00462-3.

54. Krentz, A.J.; Patel, M.B.; Bailey, C.J. New Drugs for Type 2 Diabetes Mellitus. *Drugs* **2008**, *68*, 2131–2162, doi:10.2165/00003495-200868150-00005.

55. Esposito, K.; Chiodini, P.; Bellastella, G.; Maiorino, M.I.; Giugliano, D. Proportion of Patients at HbA1c Target <7% with Eight Classes of Antidiabetic Drugs in Type 2 Diabetes: Systematic Review of 218 Randomized Controlled Trials with 78 945 Patients. *Diabetes, Obes. Metab.* **2012**, *14*, 228–233, doi:10.1111/j.1463-1326.2011.01512.x.

56. Moller DE New Drug Targets for Type 2 Diabetes and the Metabolic Syndrome. *Nature* **2001**, *414*, 821–827.

57. Chehade, J.M.; A.D., M. A Rational Approach to Drug Therapy of Type 2 Diabetes Mellitus. *Drugs* **2000**, *60*, 95–113.

58. Deans, K.A.; Sattar, N. “Anti-Inflammatory” Drugs and Their Effects on Type 2 Diabetes. *Diabetes Technol. Ther.* **2006**, *8*, 18–27, doi:10.1089/dia.2006.8.18.

59. Avery, M.; Mizuno, C.; Chittiboyina, A.; Kurtz, T.; Pershadsingh, H. Type 2 Diabetes and Oral Antihyperglycemic Drugs. *Curr. Med. Chem.* **2008**, *15*, 61–74, doi:10.2174/092986708783330656.

60. Paneni, F.; Lüscher, T.F. Cardiovascular Protection in the Treatment of Type 2 Diabetes: A Review of Clinical Trial Results Across Drug Classes. *Am. J. Cardiol.* **2017**, *120*, S17–S27, doi:10.1016/j.amjcard.2017.05.015.

61. Adeghate, E.A.; Kalász, H.; Al Jaberi, S.; Adeghate, J.; Tekes, K. Tackling Type 2 Diabetes-Associated Cardiovascular and Renal Comorbidities: A Key Challenge for Drug Development. *Expert Opin. Investig. Drugs* **2021**, *30*, 85–93, doi:10.1080/13543784.2021.1865914.

62. Gupta, R.; Walunj, S.; Tokala, R.; Parsa, K.; Singh, S.; Pal, M. Emerging Drug Candidates of Dipeptidyl Peptidase IV (DPP IV) Inhibitor Class for the Treatment of Type 2 Diabetes. *Curr. Drug Targets* **2009**, *10*, 71–87, doi:10.2174/138945009787122860.

63. Tamborlane, W. V.; Haymond, M.W.; Dunger, D.; Shankar, R.; Gubitosi-Klug, R.; Bethin, K.; Karres, J.; Tomasi, P.; Libman, I.; Hale, P.H.; et al. Expanding Treatment Options for Youth With Type 2 Diabetes: Current Problems and Proposed Solutions. *Diabetes Care* **2016**, *39*, 323–329.

64. Lu, D.-Y.; Che, J.-Y.; Yarla, N.S.; Wu, H.-Y.; Lu, T.-R.; Xu, B.; Wu, S.-Y.; Ding, J.; Lu, Y.; Zhu, H. Type 2 Diabetes Treatment and Drug Development Study. *Open Diabetes J.* **2018**, *8*, 22–33, doi:10.2174/1876524601808010022.

65. Thrasher, J. Pharmacologic Management of Type 2 Diabetes Mellitus: Available Therapies. *Am. J. Cardiol.* **2017**, *120*, S4–S16, doi:10.1016/j.amjcard.2017.05.009.

66. Kalsi A; Singh S; Taneja N; Kukal S; Mani S Current Treatments for Type 2 Diabetes, Their Side Effects and Possible Complementary Treatments. *Int. J. Pharm. Pharm. Sci.* **2015**, *7*, 13–18.

67. Olokoba, A.B.; Obateru, O.A.; Olokoba, L.B. Type 2 Diabetes Mellitus: A Review of Current Trends. *Oman Med. J.* **2012**, *27*, 269–273, doi:10.5001/omj.2012.68.

68. Kong, M.; Xie, K.; Lv, M.; Li, J.; Yao, J.; Yan, K.; Wu, X.; Xu, Y.; Ye, D. Anti-Inflammatory Phytochemicals for the Treatment of Diabetes and Its Complications: Lessons Learned and Future Promise. *Biomed. Pharmacother.* **2021**, *133*, doi:10.1016/j.biopha.2020.110975.

69. Tripathi, A.K.; Kohli, S. Anti-Diabetic Activity and Phytochemical Screening of Crude Extracts of PuerariaTuberosa DC. (FABACEAE) Grown in India on STZ -Induced Diabetic Rats. *Asian J. Med. Pharm. Res* **2013**, *3*, 66–73.

70. Hassan, M.M.; Uddin, S.; Bhowmik, A.; Ashraf, A.; Islam, M.M.; Rokeya, B. Phytochemical Screening and Antidiabetic Effects of Fruit Rind of Momordica Dioica Roxb. on Streptozocin Induced Type 2 Diabetic Rats. *Heliyon* **2022**, *8*, doi:10.1016/j.heliyon.2022.e08771.

71. Yasmin, A, Bukhari, S. A., Zahoor, M. K., Mustafa, G., & Rasul, A. Screening of Novel Phytochemicals as Secreted Frizzled-Related Protein 4 Inhibitors: An Early Stage Biomarker of Type 2 Diabetes. *Pak. J. Pharm. Sci.* **2020**, *33(3)*, 1245–1250.

72. Singh, R.; Kazmi, I.; Afzal, M.; Imam, F.; Alharbi, K.S. Dietary Phytochemicals and Their Potential Effects on Diabetes Mellitus 2. *Plant Hum. Heal. Pharmacol. Ther. Uses* **2019**, *3*, 65–86, doi:10.1007/978-3-030-04408-4_4.

73. González-Castejón, M.; Rodriguez-Casado, A. Dietary Phytochemicals and Their Potential Effects on Obesity: A Review. *Pharmacol. Res.* **2011**, *64*, 438–455, doi:10.1016/j.phrs.2011.07.004.

74. Hariftyani, A.S.; Kurniawati, L.A.; Khaerunnisa, S.; Veterini, A.S.; Setiawati, Y.; Awaluddin, R. In Silico Analysis of Potential Antidiabetic Phytochemicals from Matricaria Chamomilla l. Against Ptp1b and Aldose Reductase for Type 2 Diabetes Mellitus and Its Complications. *Nat. Prod. Sci.* **2021**, *27*, 99–114, doi:10.20307/nps.2021.27.2.99.

75. Adefegha, A. Phytochemistry and Mode of Action of Some Tropical Spices in the Management of Type-2 Diabetes and Hypertension. *African J. Pharm. Pharmacol.* **2013**, *7*, 332–346, doi:10.5897/ajppx12.014.

76. Atere, T.G.; Akinloye, O.A.; Ugbaja, R.N.; Ajiboye Ojo, D.; Samuel Metibemu, D.; Omotuyi, I. olaposi Molecular Interactions and Docking Studies of the Phytochemical Constituents of Costus Afer against Therapeutic Targets of Type 2 Diabetes. *J. Appl. Bioinforma. Comput. Biol.* **2017**, *06*, doi:10.4172/2329-9533.1000139.

77. Rampogu, S.; Parameswaran, S.; Lemuel, M.R.; Lee, K.W. Exploring the Therapeutic Ability of Fenugreek against Type 2 Diabetes and Breast Cancer Employing Molecular Docking and Molecular Dynamics Simulations. *Evidence-based Complement. Altern. Med.* **2018**, *2018*, doi:10.1155/2018/1943203.

78. Kausar, M.A.; Shahid, S.M.A.; Anwar, S.; Kuddus, M.; Khan, M.K.A.; Khalifa, A.M.; Khatoon, F.; Alotaibi, A.D.; Alkhodairy, S.F.; Snoussi, M.; et al. Identifying the Alpha-Glucosidase Inhibitory Potential of Dietary Phytochemicals against Diabetes Mellitus Type 2 via Molecular Interactions and Dynamics Simulation. *Cell. Mol. Biol.* **2021**, *67*, 16–26, doi:10.14715/CMB/2021.67.5.3.

79. Sari, Y.; Indarto, D.; Wasita, B. Identification of Epicatechin Gallate and Other Phytochemicals in Methanol Extract of Fresh and Dried Star-Fruits (Averrhoa Carambola Linn.) for Treatment of Type 2 Diabetes Mellitus. **2021**, doi:10.4108/eai.17-7-2021.2312400.

80. Onaolapo, A.Y.; Onaolapo, O.J. Nutraceuticals and Diet-Based Phytochemicals in Type 2 Diabetes Mellitus: From Whole Food to Components with Defined Roles and Mechanisms. *Curr. Diabetes Rev.* **2018**, *16*, 12–25, doi:10.2174/1573399814666181031103930.

81. Belobrajdic, D.P.; Bird, A.R. The Potential Role of Phytochemicals in Wholegrain Cereals for the Prevention of Type-2 Diabetes. *Nutr. J.* **2013**, *12*, doi:10.1186/1475-2891-12-62.

82. Oboh, G.; Olabiyi, A.A.; Akinyemi, A.J.; Ademiluyi, A.O. Inhibition of Key Enzymes Linked to Type 2 Diabetes and Sodium Nitroprusside-Induced Lipid Peroxidation in Rat Pancreas by Water-Extractable Phytochemicals from Unripe Pawpaw Fruit (Carica Papaya). *J. Basic Clin. Physiol. Pharmacol.* **2014**, *25*, 21–34, doi:10.1515/jbcpp-2013-0002.

83. Lin, Y.; Li, J.; Wu, D.; Wang, F.; Fang, Z.; Shen, G. Identification of Hub Genes in Type 2 Diabetes Mellitus Using Bioinformatics Analysis. *Diabetes, Metab. Syndr. Obes.* **2020**, *13*, 1793–1801, doi:10.2147/DMSO.S245165.

84. Zhong, M.; Wu, Y.; Ou, W.; Huang, L.; Yang, L. Identification of Key Genes Involved in Type 2 Diabetic Islet Dysfunction: A Bioinformatics Study. *Biosci. Rep.* **2019**, *39*, doi:10.1042/BSR20182172.

85. Vastrad, B.; Tengli, A.; Vastrad, C.; Kotturshetti, I. Bioinformatics Analysis of Key Genes and Pathways for Obesity Associated Type 2 Diabetes Mellitus as a Therapeutic Target. *bioRxiv* **2020**, doi:10.1101/2020.12.25.424383.

86. Vastrad, B.; Vastrad, C. Bioinformatics Analysis of Potential Key Genes and Mechanisms in Type 2 Diabetes Mellitus. *bioRxiv* **2021**, 2021.03.28.437386.

87. Ding, L.; Fan, L.; Xu, X.; Fu, J.; Xue, Y. Identification of Core Genes and Pathways in Type 2 Diabetes Mellitus by Bioinformatics Analysis. *Mol. Med. Rep.* **2019**, *20*, 2597–2608, doi:10.3892/mmr.2019.10522.

88. Prashanth, G.; Vastrad, B.; Tengli, A.; Vastrad, C.; Kotturshetti, I. Investigation of Candidate Genes and Mechanisms Underlying Obesity Associated Type 2 Diabetes Mellitus Using Bioinformatics Analysis and Screening of Small Drug Molecules. *BMC Endocr. Disord.* **2021**, *21*, doi:10.1186/s12902-021-00718-5.

89. Bioinformatics Analysis of Genes Associted With Type 2 Diabetes Mellitus.

90. Dong, Z.; Lei, X.; Kujawa, S.A.; Bolu, N.E.; Zhao, H.; Wang, C. Identification of Core Gene in Obese Type 2 Diabetes Patients Using Bioinformatics Analysis. *Adipocyte* **2021**, *10*, 310–321, doi:10.1080/21623945.2021.1933297.

91. Liu, G.; Zeng, H.; Zhang, C.; Xu, J. Pathology - Research and Practice Key Genes Associated with Diabetes Mellitus and Hepatocellular Carcinoma. *Pathol. - Res. Pract.* **2019**, *215*, 152510.

92. Che, X.; Zhao, R.; Xu, H.; Liu, X.; Zhao, S.; Ma, H. Differently Expressed Genes (Degs) Relevant to Type 2 Diabetes Mellitus Identification and Pathway Analysis via Integrated Bioinformatics Analysis. *Med. Sci. Monit.* **2019**, *25*, 9237–9244, doi:10.12659/MSM.918407.

93. Zhou, Z.; Zhu, Y.; Liu, Y.; Yin, Y. Comprehensive Transcriptomic Analysis Indicates Brain Regional Specific Alterations in Type 2 Diabetes. *Aging (Albany. NY).* **2019**, *11*, 6398–6421, doi:10.18632/aging.102196.

94. Moller, D.E. New Drug Targets for Type 2 Diabetes and the Metabolic Syndrome. *Nature* **2001**, *414*, 821–827, doi:10.1038/414821a.

95. Chehade, J.M.; Mooradian, A.D. A Rational Approach to Drug Therapy of Type 2 Diabetes Mellitus. *Drugs* **2000**, *60*, 95–113, doi:10.2165/00003495-200060010-00006.

96. Yan, X.; Wan, H.; Hao, X.; Lan, T.; Li, W.; Xu, L.; Yuan, K.; Wu, H. Importance of Gene Expression Signatures in Pancreatic Cancer Prognosis and the Establishment of a Prediction Model. *Cancer Manag. Res.* **2019**, *11*, 273–283, doi:10.2147/CMAR.S185205.

97. Li, H.; Wang, X.; Fang, Y.; Huo, Z.; Lu, X.; Zhan, X.; Deng, X.; Peng, C.; Shen, B. Integrated Expression Profiles Analysis Reveals Novel Predictive Biomarker in Pancreatic Ductal Adenocarcinoma. *Oncotarget* **2017**, *8*, 52571–52583, doi:10.18632/oncotarget.16732.

98. Wang, Y.; Li, Y. Analysis of Molecular Pathways in Pancreatic Ductal Adenocarcinomas with a Bioinformatics Approach. *Asian Pacific J. Cancer Prev.* **2015**, *16*, 2561–2567, doi:10.7314/APJCP.2015.16.6.2561.

99. Li, N.; Zhao, X.; You, S. Identification of Key Regulators of Pancreatic Ductal Adenocarcinoma Using Bioinformatics Analysis of Microarray Data. *Med. (United States)* **2019**, *98*, 1–10, doi:10.1097/MD.0000000000014074.

100. He, Y.; Liu, Y.; Gong, J.; Liu, C.; Zhang, H.; Wu, H. Identification of Key Pathways and Candidate Genes in Pancreatic Ductal Adenocarcinoma Using Bioinformatics Analysis. *Oncol. Lett.* **2019**, *17*, 3751–3764, doi:10.3892/ol.2019.10041.

101. Pahari, P.; Basak, P.; Sarkar, A. Ensemble Based Biomarker Identification on Pancreatic Ductal Adenocarcinoma Gene Expressions. *Int. J. Comput. Appl.* **2019**, *7074*, doi:10.1080/1206212X.2019.1636524.

102. Ding, J.; Liu, Y.; Lai, Y. Identifying MMP14 and COL12A1 as a Potential Combination of Prognostic Biomarkers in Pancreatic Ductal Adenocarcinoma Using Integrated Bioinformatics Analysis. *PeerJ* **2020**, *8*, 1–20, doi:10.7717/peerj.10419.

103. Cheng, Y.; Wang, K.; Geng, L.; Sun, J.; Xu, W.; Liu, D.; Gong, S.; Zhu, Y. Identification of Candidate Diagnostic and Prognostic Biomarkers for Pancreatic Carcinoma. *EBioMedicine* **2019**, *40*, 382–393, doi:10.1016/j.ebiom.2019.01.003.

104. Raman, P.; Maddipati, R.; Lim, K.H.; Tozeren, A. Pancreatic Cancer Survival Analysis Defines a Signature That Predicts Outcome. *PLoS One* **2018**, *13*, 1–18, doi:10.1371/journal.pone.0201751.

105. Zhou, Y.Y.; Chen, L.P.; Zhang, Y.; Hu, S.K.; Dong, Z.J.; Wu, M.; Chen, Q.X.; Zhuang, Z.Z.; Du, X.J. Integrated Transcriptomic Analysis Reveals Hub Genes Involved in Diagnosis and Prognosis of Pancreatic Cancer. *Mol. Med.* **2019**, *25*, 1–13, doi:10.1186/s10020-019-0113-2.

106. Zhang, J.; Yang, J.; Wang, H.; Sun, H.; Fang, Y.; Liu, S.; Zhao, Z.; Qin, H.; Yang, J.; Huang, W.; et al. Alteration of Tumor-Associated Macrophage Subtypes Mediated by KRT6A in Pancreatic Ductal Adenocarcinoma. *Aging (Albany. NY).* **2020**, *12*, 23217–23231, doi:10.18632/aging.104091.

107. Zhou, J.; Hui, X.; Mao, Y.; Fan, L. Identification of Novel Genes Associated with a Poor Prognosis in Pancreatic Ductal Adenocarcinoma via a Bioinformatics Analysis. *Biosci. Rep.* **2019**, *39*, 1–16, doi:10.1042/BSR20190625.

108. Long, J.; Liu, Z.; Wu, X.; Xu, Y.; Ge, C. Gene Expression Profile Analysis of Pancreatic Cancer Based on Microarray Data. *Mol. Med. Rep.* **2016**, *13*, 3913–3919, doi:10.3892/mmr.2016.5021.

109. Jin, D.; Jiao, Y.; Ji, J.; Jiang, W.; Ni, W.; Wu, Y.; Ni, R.; Lu, C.; Qu, L.; Ni, H.; et al. Identification of Prognostic Risk Factors for Pancreatic Cancer Using Bioinformatics Analysis. *PeerJ* **2020**, *2020*, 1–22, doi:10.7717/peerj.9301.

110. Li, C.; Zeng, X.; Yu, H.; Gu, Y.; Zhang, W. Identification of Hub Genes with Diagnostic Values in Pancreatic Cancer by Bioinformatics Analyses and Supervised Learning Methods. *World J. Surg. Oncol.* **2018**, *16*, 1–12, doi:10.1186/s12957-018-1519-y.

111. Sun, D.; Jin, H.; Zhang, J.; Tan, X. Integrated Whole Genome Microarray Analysis and Immunohistochemical Assay Identifies COL11A1, GJB2 and CTRL as Predictive Biomarkers for Pancreatic Cancer. *Cancer Cell Int.* **2018**, *18*, 1–11, doi:10.1186/s12935-018-0669-x.

112. Liu, J.; Li, J.; Li, H.; Li, A.; Liu, B.; Han, L. A Comprehensive Analysis of Candidate Genes and Pathways in Pancreatic Cancer. *Tumor Biol.* **2015**, *36*, 1849–1857, doi:10.1007/s13277-014-2787-y.

113. Wang, Y.; Liu, K.; Ma, Q.; Tan, Y.; Du, W.; Lv, Y.; Tian, Y.; Wang, H. Pancreatic Cancer Biomarker Detection by Two Support Vector Strategies for Recursive Feature Elimination. *Biomark. Med.* **2019**, *13*, 105–121, doi:10.2217/bmm-2018-0273.

114. Bhasin, M.K.; Ndebele, K.; Bucur, O.; Yee, E.U.; Otu, H.H.; Plati, J.; Bullock, A.; Gu, X.; Castan, E.; Zhang, P.; et al. Meta-Analysis of Transcriptome Data Identifies a Novel 5-Gene Pancreatic Adenocarcinoma Classifier. *Oncotarget* **2016**, *7*, 23263–23281, doi:10.18632/oncotarget.8139.

115. Qiu, X.; Hou, Q.H.; Shi, Q.Y.; Jiang, H.X.; Qin, S.Y. Identification of Hub Prognosis-Associated Oxidative Stress Genes in Pancreatic Cancer Using Integrated Bioinformatics Analysis. *Front. Genet.* **2020**, *11*, doi:10.3389/fgene.2020.595361.

116. Shang, M.; Zhang, L.; Chen, X.; Zheng, S. Identification of Hub Genes and Regulators Associated with Pancreatic Ductal Adenocarcinoma Based on Integrated Gene Expression Profile Analysis. *Discov. Med.* **2019**, *28*, 159–172.

117. Ma, Y.; Pu, Y.; Peng, L.; Luo, X.; Xu, J.; Peng, Y.; Tang, X. Identification of Potential Hub Genes Associated with the Pathogenesis and Prognosis of Pancreatic Duct Adenocarcinoma Using Bioinformatics Meta‑analysis of Multi‑platform Datasets. *Oncol. Lett.* **2019**, *18*, 6741–6751, doi:10.3892/ol.2019.11042.

118. Liu, H.; Zhou, Q.; Wei, W.; Qi, B.; Zeng, F.; Bao, N.; Li, Q.; Guo, F.; Xia, S. The Potential Drug for Treatment in Pancreatic Adenocarcinoma: A Bioinformatical Study Based on Distinct Drug Databases. *Chinese Med. (United Kingdom)* **2020**, *15*, 1–13, doi:10.1186/s13020-020-00309-x.

119. Islam, S.; Kitagawa, T.; Baron, B.; Abiko, Y.; Chiba, I.; Kuramitsu, Y. ITGA2, LAMB3, and LAMC2 May Be the Potential Therapeutic Targets in Pancreatic Ductal Adenocarcinoma: An Integrated Bioinformatics Analysis. *Sci. Rep.* **2021**, *11*, doi:10.1038/s41598-021-90077-x.

120. Wu, J.; Liu, J.; Wei, X.Q.; Yu, Q.; Niu, X.H.; Tang, S.H.; Song, L. A Feature-Based Analysis Identifies COL1A2 as a Regulator in Pancreatic Cancer. *J. Enzyme Inhib. Med. Chem.* **2019**, *34*, doi:10.1080/14756366.2018.1484734.

121. Topalovski, M.; Brekken, R.A. Matrix Control of Pancreatic Cancer: New Insights into Fibronectin Signaling. *Cancer Lett.* 2016, *381*.

122. Hu, D.; Ansari, D.; Pawlowski, K.; Zhou, Q.; Sasor, A.; Welinder, C.; Kristl, T.; Bauden, M.; Rezeli, M.; Jiang, Y.; et al. Proteomic Analyses Identify Prognostic Biomarkers for Pancreatic Ductal Adenocarcinoma. *Oncotarget* **2018**, *9*, doi:10.18632/oncotarget.23929.

123. Lu, W.; Fei, A.; Jiang, Y.; Chen, L.; Wang, Y. Tetraspanin CD9 Interacts with α-Secretase to Enhance Its Oncogenic Function in Pancreatic Cancer. *Am. J. Transl. Res.* **2020**, *12*, 5525–5537.

124. Mueller, A.C.; Piper, M.; Goodspeed, A.; Bhuvane, S.; Williams, J.S.; Bhatia, S.; Phan, A. V.; Van Court, B.; Zolman, K.L.; Peña, B.; et al. Induction of ADAM10 by RT Drives Fibrosis, Resistance, and EMT in Pancreatic Cancer. *Cancer Res.* **2021**.

125. Iwatate, Y.; Yokota, H.; Hoshino, I.; Ishige, F.; Kuwayama, N.; Itami, M.; Mori, Y.; Chiba, S.; Arimitsu, H.; Yanagibashi, H.; et al. Transcriptomic Analysis Reveals High ITGB1 Expression as a Predictor for Poor Prognosis of Pancreatic Cancer. *PLoS One* **2022**, *17*, doi:10.1371/journal.pone.0268630.

126. Puzanov, G.A. Identification of Key Genes of the CcRCC Subtype with Poor Prognosis. *Sci. Rep.* **2022**, 1–10, doi:10.1038/s41598-022-18620-y.

127. Wu, P.; Xiang, T.; Wang, J.; Lv, R.; Wu, G. TYROBP Is a Potential Prognostic Biomarker of Clear Cell Renal Cell Carcinoma. **2020**, *10*, 2588–2604, doi:10.1002/2211-5463.12993.

128. Zhao, E. Identification of Key Genes of Prognostic Value in Clear Cell Renal Cell Carcinoma Microenvironment and a Risk Score Prognostic Model. **2020**, *2020*.

129. Tian, Z.; Yuan, C.; Yang, K.; Gao, X. Systematic Identification of Key Genes and Pathways in Clear Cell Renal Cell Carcinoma on Bioinformatics Analysis. **2019**, *7*, doi:10.21037/atm.2019.01.18.

130. Peng, L.; Cao, Z.; Wang, Q.; Fang, L.; Yan, S.; Xia, D.; Wang, J.; Bi, L. Screening of Possible Biomarkers and Therapeutic Targets in Kidney Renal Clear Cell Carcinoma: Evidence from Bioinformatic Analysis. *Front. Oncol.* **2022**, *12*, 1–10, doi:10.3389/fonc.2022.963483.

131. Yuan, L.; Zeng, G.; Chen, L.; Wang, G.; Wang, X.; Cao, X. Identification of Key Genes and Pathways in Human Clear Cell Renal Cell Carcinoma ( CcRCC ) by Co-Expression Analysis. **2018**, *14*, doi:10.7150/ijbs.23574.

132. Ma, S.; Ge, Y.; Xiong, Z.; Wang, Y.; Li, L.; Chao, Z. A Novel Gene Signature Related to Oxidative Stress Predicts the Prognosis in Clear Cell Renal Cell Carcinoma. **2023**, 1–22, doi:10.7717/peerj.14784.

133. Zhai, W.; Lu, H.; Dong, S.; Fang, J.; Yu, Z. Identification of Potential Key Genes and Key Pathways Related to Clear Cell Renal Cell Carcinoma through Bioinformatics Analysis. *Acta Biochim. Biophys. Sin. (Shanghai).* **2020**, *52*, 853–863, doi:10.1093/abbs/gmaa068.

134. Zhong, T.; Wang, X.; Wang, H. Key Genes Associated With Prognosis and Metastasis of Clear Cell Renal Cell Carcinoma. **2021**, 1–17.

135. Peng, R. Identification of Core Genes Involved in the Metastasis of Clear Cell Renal Cell Carcinoma. **2020**, 13437–13449.

136. Peng, Y.; Dong, S.; Wang, H. Key Sunitinib- ­ Related Biomarkers for Renal Cell Carcinoma. **2021**, 6917–6930, doi:10.1002/cam4.4206.

137. Hu, J.; Chen, Z.; Bao, L.; Zhou, L.; Hou, Y.; Liu, L.; Xiong, M.; Zhang, Y.; Wang, B.; Tao, Z.; et al. Single-Cell Transcriptome Analysis Reveals Intratumoral Heterogeneity in CcRCC , Which Results in Different Clinical Outcomes. *Mol. Ther.* **2020**, *28*, 1658–1672, doi:10.1016/j.ymthe.2020.04.023.

138. Huang, H.; Zhu, L.; Huang, C.; Dong, Y.; Fan, L.; Tao, L.; Peng, Z.; Xiang, R. Identification of Hub Genes Associated With Clear Cell Renal Cell Carcinoma by Integrated Bioinformatics Analysis. *Front. Oncol.* **2021**, *11*, 1–12, doi:10.3389/fonc.2021.726655.

139. Luo, T.; Chen, X.; Zeng, S.; Guan, B.; Hu, B.O.; Meng, Y.U.; Liu, F.; Wong, T.; Lu, Y.; Yun, C.; et al. Bioinformatic Identification of Key Genes and Analysis of Prognostic Values in Clear Cell Renal Cell Carcinoma. *Oncol. Lett.* **2018**, *16*, 1747–1757, doi:10.3892/ol.2018.8842.

140. Zhang, Y.; Chen, M.; Liu, M.; Xu, Y.; Wu, G. Glycolysis-Related Genes Serve as Potential Prognostic Biomarkers in Clear Cell Renal Cell Carcinoma. **2021**, *2021*.

141. Bao, L.; Zhao, Y.; Liu, C.; Cao, Q.; Huang, Y.; Chen, K.; Song, Z. The Identification of Key Gene Expression Signature and Biological Pathways in Metastatic Renal Cell Carcinoma. **2020**, *11*, doi:10.7150/jca.38379.

142. Wei, X.; Deng, W.; Dong, Z.; Luo, Y.; Hu, X.; Zhang, J.; Xie, Z.; Zheng, T.; Tan, Y.; Tang, Z.; et al. Redox Metabolism-Associated Molecular Classification of Clear Cell Renal Cell Carcinoma. **2022**, *2022*.

143. Gu, D. Identifying the Novel Key Genes in Renal Cell Carcinoma by Bioinformatics Analysis and Cell Experiments CURRENT STATUS : UNDER REVIEW., doi:10.21203/rs.2.23818/v1.

144. Zhang, Q.; Ding, L.; Zhou, T.; Zhai, Q.; Ni, C. A Metabolic Reprogramming- Related Prognostic Risk Model for Clear Cell Renal Cell Carcinoma : From Construction to Preliminary Application. **2022**, 1–15, doi:10.3389/fonc.2022.982426.

145. Yuan, L.; Chen, L.; Qian, K.; Qian, G.; Wu, C.L.; Wang, X.; Xiao, Y. Co-Expression Network Analysis Identified Six Hub Genes in Association with Progression and Prognosis in Human Clear Cell Renal Cell Carcinoma (CcRCC). *Genomics Data* **2017**, *14*, 132–140, doi:10.1016/j.gdata.2017.10.006.

146. Liu, Y.; Huang, Z.; Cheng, G.; Shou, Y.; Xu, J.; Liu, D.; Yang, H.; Liang, H.; Zhang, X. Development of a Four-Gene Prognostic Model for Clear Cell Renal Cell Carcinoma Based on Transcriptome Analysis. *Genomics* **2021**, *113*, 1816–1827, doi:10.1016/j.ygeno.2021.04.005.

147. Zhang, Z.; Lin, E.; Zhuang, H.; Xie, L.; Feng, X.; Liu, J.; Yu, Y. Construction of a Novel Gene ‑ Based Model for Prognosis Prediction of Clear Cell Renal Cell Carcinoma. *Cancer Cell Int.* **2020**, 1–18, doi:10.1186/s12935-020-1113-6.

148. Yu, X.; Wu, H.; Wang, H.; Dong, H.; Gao, B. Identification of 8 Feature Genes Related to Clear Cell Renal Cell Carcinoma Progression Based on Co-Expression Analysis. **2022**, 113–124, doi:10.1159/000520832.

149. Li, R.; Wang, L.E.I.; Wang, X.; Geng, R.X.I.N.; Li, N.; Liu, X.I.U.H. Identification of Hub Genes Associated with Outcome of Clear Cell Renal Cell Carcinoma. **2020**, 2846–2860, doi:10.3892/ol.2020.11389.

150. Sui, Y.; Lu, K.; Fu, L. Prediction and Analysis of Novel Key Genes ITGAX, LAPTM5, SERPINE1 in Clear Cell Renal Cell Carcinoma through Bioinformatics Analysis. *PeerJ* **2021**, *9*, 1–21, doi:10.7717/peerj.11272.

151. Yuan, Y.; Wang, J.; Huang, L.; Guo, Y. Bioinformatics Identification of Prognostic Genes and Potential Interaction Analysis in Renal Cell Carcinoma. **2023**, doi:10.21037/tcr-22-2242.

152. Wang, S.; Yu, Z.H.; Chai, K.Q. Identification of CFTR as a Novel Key Gene in Chromophobe Renal Cell Carcinoma through Bioinformatics Analysis. *Oncol. Lett.* **2019**, *18*, 1767–1774, doi:10.3892/ol.2019.10476.

153. Sanders, E.; Diehl, S. Analysis and Interpretation of Transcriptomic Data Obtained from Extended Warburg Effect Genes in Patients with Clear Cell Renal Cell Carcinoma. *Oncoscience* **2015**, *2*, 151–186, doi:10.18632/oncoscience.128.

154. Li, K.; Zhu, Y.; Cheng, J.; Li, A.; Liu, Y.; Yang, X.; Huang, H.; Peng, Z.; Xu, H. A Novel Lipid Metabolism Gene Signature for Clear Cell Renal Cell Carcinoma Using Integrated Bioinformatics Analysis. *Front. Cell Dev. Biol.* **2023**, *11*, 1–14, doi:10.3389/fcell.2023.1078759.
